# Targeted Deletion of Kindlin-2 in Mouse Mammary Glands Inhibits Tumor Growth, Invasion, and Metastasis Downstream of a TGF-β/EGF Oncogenic Signaling Pathway

**DOI:** 10.3390/cancers14030639

**Published:** 2022-01-27

**Authors:** Wei Wang, Priyanka S. Rana, Akram Alkrekshi, Katarzyna Bialkowska, Vesna Markovic, William P. Schiemann, Edward F. Plow, Elzbieta Pluskota, Khalid Sossey-Alaoui

**Affiliations:** 1 Department of Medicine, Case Western Reserve University, Cleveland, OH 44106, USA; wxw363@case.edu (W.W.); pxr240@case.edu (P.S.R.); axa1061@case.edu (A.A.); 2 Department of Medicine, MetroHealth Medical Center, Cleveland, OH 44109, USA; vmarkovic@metrohealth.org; 3 Department of Cardiovascular and Metabolic Sciences, Lerner Research Institute, Cleveland Clinic, Cleveland, OH 44195, USA; bialkok@ccf.org (K.B.); plowe@ccf.org (E.F.P.); 4 Case Comprehensive Cancer Center, Case Western Reserve University, Cleveland, OH 44106, USA; wps20@case.edu

**Keywords:** Kindlin-2, mammary epithelial cells, breast cancer, tissue specific mouse knockout, TGF-β

## Abstract

**Simple Summary:**

Studies from our group and others have established Kindlin-2 that is expressed in the cancer cells as a major driver of tumor progression and metastasis in breast cancer (BC). The role of Kindlin-2 that is expressed in the mammary glands (tumor microenvironment) in the pathogenesis of BC has, however, not been investigated. To this end, we generated a mouse strain that specifically lacks expression of Kindlin-2 in the basal cells within the mouse mammary glands. Loss of Kindlin-2 in this mammary gland compartment, while having no effect on mouse development, significantly inhibited BC tumors growth and metastasis when these Kindlin-2-deficient mice were challenged with mammary fat pad injection of cancer cells. At the molecular signaling level, we found that Kindlin-2 plays a significant role in regulating BC progression and metastasis in both the cancer cells and the tumor microenvironment (mammary epithelial cells) downstream of a TGF-β/EGF signaling axis.

**Abstract:**

Breast cancer (BC) is one of the leading causes of cancer-related deaths due in part to its invasive and metastatic properties. Kindlin-2 (FERMT2) is associated with the pathogenesis of several cancers. Although the role of Kindlin-2 in regulating the invasion-metastasis cascade in BC is widely documented, its function in BC initiation and progression remains to be fully elucidated. Accordingly, we generated a floxed mouse strain by targeting the *Fermt2* (K2^lox/lox^) locus, followed by tissue-specific deletion of Kindlin-2 in the myoepithelial compartment of the mammary glands by crossing the K2^lox/lox^ mice with K14-Cre mice. Loss of Kindlin-2 in mammary epithelial cells (MECs) showed no deleterious effects on mammary gland development, fertility, and lactation in mice bearing Kindlin-2-deletion. However, in a syngeneic mouse model of BC, mammary gland, specific knockout of Kindlin-2 inhibited the growth and metastasis of murine E0771 BC cells inoculated into the mammary fat pads. However, injecting the E0771 cells into the lateral tail vein of Kindlin-2-deleted mice had no effect on tumor colonization in the lungs, thereby establishing a critical role of MEC Kindlin-2 in supporting BC tumor growth and metastasis. Mechanistically, we found the MEC Kindlin-2-mediated inhibition of tumor growth and metastasis is accomplished through its regulation of the TGF-β/ERK MAP kinase signaling axis. Thus, Kindlin-2 within the mammary gland microenvironment facilitates the progression and metastasis of BC.

## 1. Introduction

Kindlin-2 belongs to a three-member FERM domain protein family (Kindlin-1-3), which are encoded by the *FERMT1-3* genes. The human *FERMT1* gene (Kindlin-1) is located on chromosome 20p12.3, while its murine orthologue *Fermt1* maps to mouse chromosome 2. Human *FERMT2* (Kindlin-2) is located on chromosome 14q22.1, and mouse *Fermt2* maps to chromosome 14. Finally, the human *FERMT3* gene (Kindlin-3) maps to chromosome 11q13.1 and its murine counterpart is located on chromosome 19. Each of the three mammalian Kindlins contains a FERM domain composed of F1, F2, and F3 subdomains preceded by a F0 subdomain, and is distinguished from other FERM proteins by the presence of a PH domain that splits the F2 subdomain. Together with other lipid-binding subdomains, the PH domain facilitates targeting of Kindlins to membranes [1,2] and the interactions and activation of integrins [3,4]. While the DNA sequences of the three Kindlin family members are more than 50% identical [3,5,6], each has distinct functions. Kindlin-1 and -3 appear to have evolved from duplication of a primordial Kindlin-2 gene, which retained the ancestral features of the FERMT genes. Variable sequence regions that are interspersed among conserved regions may be responsible for imparting the distinct functions to the Kindlins [7]. In mouse studies, global genome inactivation of the Kindlin-2 gene leads to embryonic lethality as early as day 7.5 as a result of defects in heart development [6,8,9,10] and blood-vessel formation and integrity [11,12], as well as lesions in osteogenesis [13]. On the other hand, postnatal deletion of Kindlin-2 is associated with fibrosis, delayed wound healing, angiogenic defects, and heart rupture [14]. Interestingly, hemizygous deletion of Kindlin-2 in mice supports normal development and an absence of an overt phenotype, but when these mice are challenged (e.g., by aortic constriction), defects become apparent in K2^+/−^ mice attributable to defective activation of certain integrins [11], support of clathrin-dependent cell surface receptor recycling and hemostasis [15], and maintenance of vascular integrity, a consequence of disorganization of adherens junctions [16]. Deficiencies of K1 and K3 also have profound pathological consequences. For instance, K1-deficiency gives rise to a skin-fragility disorder called Kindler Syndrome, while K3-deficiency results in LAD III and a susceptibility to bleeding, infections, and bone abnormalities [6,8,17,18,19].

Recently, targeted inactivation of Kindlin-2 expression has been shown to inhibit carcinoma cell migration, invasion, and metastasis in several cancers of epithelial origin (reviewed in [20,21,22]). Our published studies established that Kindlin-2 can promote the invasion-metastasis cascade in breast cancer (BC) by controlling several hallmarks of cancer [20,21,23,24,25]. We have also shown that Kindlin-2 in cancer cells plays a major role in regulating the interactions between BC tumors and their microenvironment [25]. However, the relative contribution of Kindlin-2 during mammary gland development, as well as during the malignant transformation of mammary epithelial cells and their eventual progression to metastasis, remains to be elucidated. Here, we used a CRISPR/Cas9-based editing strategy to generate a floxed mouse in the *Fermt2* (K2^lox/lox^) locus, followed by tissue-specific deletion of Kindlin-2 in basal cells of mouse mammary glands by crossing the K2^lox/lox^ mice with K14-Cre mice, a widely used model to achieve gene deletion in mammary epithelial cells. Loss of Kindlin-2 in the mammary glands had no effect on the development of mammary glands, nor did it affect fertility or lactation of mice bearing the K2-deletion phenotype and their progeny. However, in a syngeneic mouse model of BC, we showed that conditional loss of Kindlin-2 in mammary epithelial cells significantly inhibited tumor growth and metastasis when E0771 mouse BC cancer cells were inoculated in the mammary fat pads. Further, we demonstrated that inhibition of tumor growth in the primary site was specific to the loss of Kindlin-2 expression in mammary glands since injection of the E0771 cells via the tail veins showed no difference in lung and liver metastasis between Kindlin-2-deleted and wild-type mice. Mechanistically, we showed that loss of Kindlin-2 in the mammary epithelial cells inhibited both TGF-β and EGF signaling.

## 2. Materials and Methods

### 2.1. Ethics Statement

This study used C57BL/6J mice to generate the Kindlin-2 floxed mice. The K14-Cre mice (B6N.Cg-Tg(KRT14-Cre)1Amc/J; Stock number 018964) were purchased from Jackson Laboratory (Farmington, CT, USA). For the mammary gland inoculation and tail-vein injection of E0771 cells, we used 6- to 8-week-old female C57BL/6J mice, also purchased from Jackson Laboratory. All animal procedures were performed in accordance with the guidelines and regulations set and approved by the MetroHealth System, Cleveland Clinic, and NIH.

### 2.2. Cell Lines and Reagents

E0771 BC cells were obtained from American Type Culture Collection (ATCC) and maintained according the manufacturer’s protocols. The HML2 cells were kindly provided by Drs. Pollard and Kitamura [26]. The HML2 cell line was derived from the E0771 cells by serial propagation of the E0771-derived tumors in recipient mice [26]. Cells were also routinely authenticated by STR DNA fingerprinting analysis. Kindlin-2-KO cells were generated by lentiviral transduction using CRISPR/Cas9 gene editing as described [23,24,25]. We used two independent and verified Kindlin-2-specific sgRNAs for each of the human and mouse Kindlin-2 and a scrambled sgRNA (i.e., nonsilencing sgRNA, [23,24,25]). Loss of Kindlin-2 expression was verified by Western blot. For stimulation of cells with growth factors, cells were serum starved in serum-free DMEM growth medium without antibiotics overnight. The next day, the cells were stimulated with either 100 ng/mL EGF (Millipore) or 5 ng/mL TGF-β for 30 min. EGFR inhibitor ZD1839 and TGF-β receptor inhibitor SB431542 were obtained from SelleckChem and used at a concentration of 10 µM for 2 h. Gel electrophoresis reagents were from Bio-Rad.

### 2.3. Generation of the Fermt2 Conditional Knockout Mouse Strain

**Figure 1:**
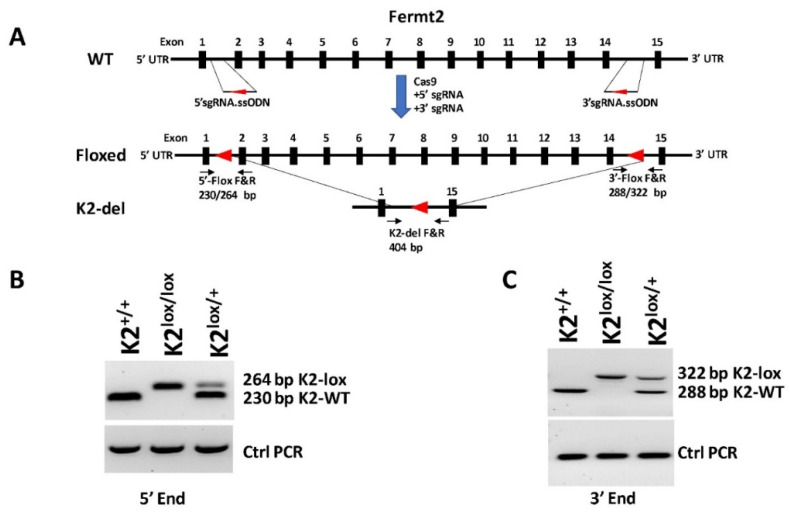
Generation of the *Fermt2* floxed mouse. (**A**) Genomic structure of the mouse *Fermt2* locus (upper panel), showing the exons and introns (not to scale). Floxed *Fermt2* was generated using CRISPR/Cas9 gene editing and homologous recombination to insert loxp sites (red triangles) in intron 1 and intron 14 (middle panel) using a 5′sgRNA.ssODN and a 3′sgRNA.ssODN (small guide RNA.single-stranded oligodeoxynucleotide donors), respectively. Oligonucleotide primer pairs 5′-Flox F&R and 3′-Flox F&R (dark arrows) were used for genotyping the ear snips using standard PCR assays to generate the PCR products with the expected sizes. Crossing of the Fermt2 Floxed mouse with a mouse expressing Cre recombinase led to the deletion of the genomic sequence between the two loxp sites, resulting in a PCR product of ~404 bp (lower panel). (**B**,**C**) Representative agarose gel pictograms showing the PCR products of ear snip DNA from wildtype (K2^+/+^), floxed Fermt2 (K2^lox/lox^), and heterozygous (K2^lox/+^) mice using the 5′-Flox F&R (**B**) and the 3′-Flox F&R (**C**) primer pairs. The expected size of the PCR products is shown. Uncropped PCR agarose gels are shown in Figure S2. Sequences of the sgRNAs and ssODNs are described in File S1 and sequences of the genotyping primers are listed in Table 1.

The *Fermt2* floxed mouse was generated by Applied StemCell using CRISPR/Cas9 gene editing and homologous recombination strategies. The step-by-step procedure is detailed in the reports provided by the company (File S1) and is summarized in Figure 1. Sequence and list of the sgRNAs are shown in File S1, and the genotyping primers are shown in Table 1. DNA preparation, PCR amplification, and agarose gel electrophoresis were performed using standard procedures.

### 2.4. Primary Tumor Growth and Metastasis Assays

Parental cells transfected with scrambled sgRNA (Control) or Kindlin-2-deficient E0771 cells (100,000 cells per mouse, *n* = 5) were implanted into the mammary fat pads in both sides (*n* = 10 tumors) of female C57BL/6J mice. Tumor growth was followed by twice-weekly monitoring of tumor volume with digital Vernier calipers. For the lung colonization assay, cells (100,000) suspended in 0.15 mL of sterile PBS were injected using a 28-gauge needle into a tail vein of six- to eight-weeks-old female C57BL/6J mice. Mice were sacrificed 5 weeks later, and the recovered lungs were fixed with 4% paraformaldehyde. Lung metastasis nodules were counted visually under a magnifying glass, and the results were plotted as the average number of metastatic foci per lobe.

### 2.5. Mammary Glands Whole Mounts

Mammary glands were isolated from the fourth inguinal glands, dissected at the indicated gestational periods, and spread on a glass slide. After fixation for 24 h in Carnoy’s fixative, the tissues were stained in carmine alum overnight [27]. Slides were then dehydrated, cleared in xylene for 5 days, and mounted in Permount mounting medium (Fisher Chemical). The mammary gland branches and the terminal end buds of the mammary glands were quantified using ImageJ software.

### 2.6. Isolation of Mammary Epithelial Cells

Mammary epithelial cells (MECs) were isolated from mammary glands of the wild-type and K2-KO mice as per the company’s guidelines (Stemcell Technologies, Inc., Vancouver, BC, Canada). In brief, the whole mammary glands were aseptically resected and transferred to the dissociation solution containing 1 part of Gentle Collagenase/Hyaluronidase (Catalog #07919) and 9 parts of complete EpiCult-B Medium (Mouse) supplemented with rh EGF (Catalog #02633), rh bFGF (Catalog #02634), and Heparin (Catalog #07980) and 10% FBS, overnight in a 37 °C water bath. Dissociated tissues were then centrifuged 350× *g* for 5 min and the pellets were resuspended in a 1:4 mixture of cold Hanks’ Balanced Salt Solution Modified (Catalog #37150) supplemented with 2% FBS and Ammonium Chloride Solution (Catalog #07800) and centrifuged at 350× *g* for 5 min. The resultant pellets containing epithelial cell organoids as well as stromal cells and lymphocytes were subjected to a further treatment to generate a single-cell suspension of mammary epithelial cells. The pellets were treated with pre-warmed Trypsin-EDTA followed by repeated pipetting for 1–3 min. 10 mL of cold Hanks’ Balanced Salt Solution Modified (Catalog #37150) supplemented with 2% FBS was added to the dissociated pellet and centrifuged at 350× *g* for 5 min. The resultant pellets were resuspended with pre-warmed Dispase (5 U/mL; Catalog #07913) and 200 µL of DNase I Solution (1 mg/mL; Catalog #07900) followed by repeated pipetting for 1 min. The cell suspension was further diluted with an additional 10 mL of the Hanks’ + FBS solution and filtered through a 40-µm Cell Strainer (Catalog #27305) into a new 50-mL centrifuge tube and later centrifuged at 350× *g* for 5 min. The pellet-containing mouse mammary epithelial cells were seeded into a culture dish in complete EpiCult-B Medium (Mouse) supplemented with rh EGF (Catalog #02633), rh bFGF (Catalog #02634) and Heparin (Catalog #07980), and 10% FBS. After 24 h the culture medium was replaced with serum-free Complete EpiCult™-B Medium (Mouse) containing cytokines. The MECs were further sub-cultured, reseeded, and frozen for further use.

### 2.7. Immunofluorescence Assays and Confocal Microscopy Analyses

For immunofluorescence, mammary epithelial cells were transferred no later than 24 h before the experiment onto No. 1.5 coverslips (Electron Microscopy Sciences, Hatfield, PA, USA, catalog # 72222-01), precoated with 0.01% poly-L-Lysine (Sigma-Aldrich, catalog # 259688-63-0). Cell cultures were maintained at 37 °C in a humidified incubator in an atmosphere of 5% CO_2_. For immunofluorescence, cells were processed as previously described [28,29]. In brief, MECs were fixed with 4% paraformaldehyde in PBS for 20 min at room temperature followed by three PBS washes. The cells were then permeabilized with 0.5% Triton in PBS for 10 min followed by three PBS washes. Next, the cells were blocked with 5% donkey serum (Jackson ImmunoResearch Laboratories, INC, West Grove, PA, USA, catalog # 017-000-121) for 60 min and stained with primary antibodies diluted in donkey serum overnight at 4 °C. The following day, the cells were washed with PBS and stained with the secondary antibodies for 1–2 h at room temperature, washed again, and mounted on slides using the ProLong Gold antifade reagent with DAPI (Invitrogen, catalog # P36931). The following antibodies were used. Mouse mAb against Kindlin-2 (1:200, Millipore Sigma, Burlington, MA, USA, catalog # MAB2617); mouse mAb against cytokeratin 14 (1:100, Invitrogen, Waltham, MA, USA, catalog # MA5-11599), rabbit mAbs against cytokeratin-14 (1:1000, Abcam, Cambridge, UK, catalog # ab181595), and against cytokeratin 8 (1:100, Abcam, Cambridge, UK, catalog # ab53280). Donkey anti-Mouse IgG (H+L) Secondary Antibody, Alexa Fluor 594-conjugated (1:2000) and Donkey anti-rabbit IgG (H+L) Secondary Antibody, Alexa Fluor 488 (1:2000) were from Invitrogen. For Confocal Microscopy analyses, images were collected with a Leica DM5500 CS using LAS X software by Leica microsystems. Images were captured with a 40× oil immersion objective lens. True magnifications are indicated in the figures by scale bars. Imaging data sets were exported to ImageJ software where montages of representative data were generated.

### 2.8. Three-Dimensional Tumorsphere Growth and Invasion Assays

The 3D tumorsphere cultures for both single and multisphere growth as well as tumorsphere invasion were performed as previously described [30,31]. Briefly, for 3D single-spheroid formation, E0771 cells or their derivatives were seeded in a 96-well ultra-low attachment (ULA) plate at a density of 1.5 × 10^3^ cells per well and centrifuged for 10 min at 125× *g* at room temperature. The plate was then incubated at 37 °C and imaged at day 3 and day 7 with a Leica DMi1 microscope to monitor the 3D single spheroid formation. For 3D spheroid invasion, E0771 cells or their derivatives were seeded in a 96-well ULA plate at a density of 1.5 × 10^3^ cells per well and centrifuged for 10 min at 125× *g* at room temperature. For assessing for the invasive potential of the spheroids, 3 days post-formation of tumorsphere, each well was supplemented with 90 µL Matrigel (1:1 in complete culture medium) on top. The plate was then incubated at 37 °C and imaged at day 3 and day 7 with a Leica DMi1 microscope to monitor the 3D spheroid invasion. For 3D multi-spheroid formation, a 6-well plate was coated with polyhema overnight to allow polymerization. The following day, cells were plated at a density of 2 × 10^3^ on each precoated well and imaged every 72 h for 11 days to monitor multi-spheroid formation with a Leica DMi1 microscope.

### 2.9. Antibodies

The following primary antibodies used for Western Blot analyses were from Cell Signaling technology, at a dilution of (1:1000): rabbit anti pERK1/2, rabbit anti ERK1/2, rabbit anti Keratin 5. Rabbit anti Kindlin-1 (1:1000), mouse anti Kindlin-2 (1:1000), and rabbit anti Kindlin-3 (1:1000) were obtained from ProCsi, and rabbit anti-pSMAD3 (1:1000) and rabbit anti-SMAD3 (1:1000) were obtained from Abcam; mouse anti-Vimentin (1:2000) was obtained from BD Pharmingen, and mouse monoclonal anti β-actin was obtained from Sigma-Aldrich (1:5000). Secondary antibodies—goat horseradish-peroxidase-conjugated anti-mouse IgG and goat horseradish-peroxidase-conjugated anti-rabbit IgG (1:2000)—were ordered from BioRad. The antibodies were dissolved to a working concentration either in 5% BSA (primary antibodies) or 5% Non-Fat Dry Milk (secondary antibodies).

### 2.10. Statistical Analyses

Experiments were performed in triplicate and analyzed using the Student’s *t*-test. In calculating two-tailed significance levels for equality of means, equal variances were assumed for the two populations. Results were considered significant at *p* < 0.05.

## 3. Results

### 3.1. Generation of the Fermt2 Floxed Mouse

The Fermt2 floxed mouse was generated by Applied StemCell Inc using CRISPR/Cas9 and a homologous recombination repair-mediated gene editing strategy as described in Figure 1. A mixture of a 5′ sgRNA + 5′ ssOND that targets intron 1, and a 3′ sgRNA + 3′ ssOND that targets intron 14, along with Cas9 (Figure 1A), was injected into C57BL/6 embryos, and the embryos were subsequently implanted into C57BL/6 pseudo-pregnant foster mothers. The resulting pups were genotyped for insertion of the loxP sequences at the desired location by PCR analyses using the PCR amplification of both the 5′ Flox and the 3′Flox amplicons (Figure 1B,C). The correct insertion of the loxP sequence resulted in a PCR product that was 34-bp larger than the wild-type sequence at the 5′ (Figure 1B) and 3′ (Figure 1C) insertion sites. The resultant PCR products were also sequenced for further validation. Mice with both 5′ loxP and 3′ loxP sites were crossed with K14-Cre mice to generate tissue-specific deletion of Fermt2.

### 3.2. Tissue Specific Deletion of Fermt2 in Mammary Glands

**Figure 2:**
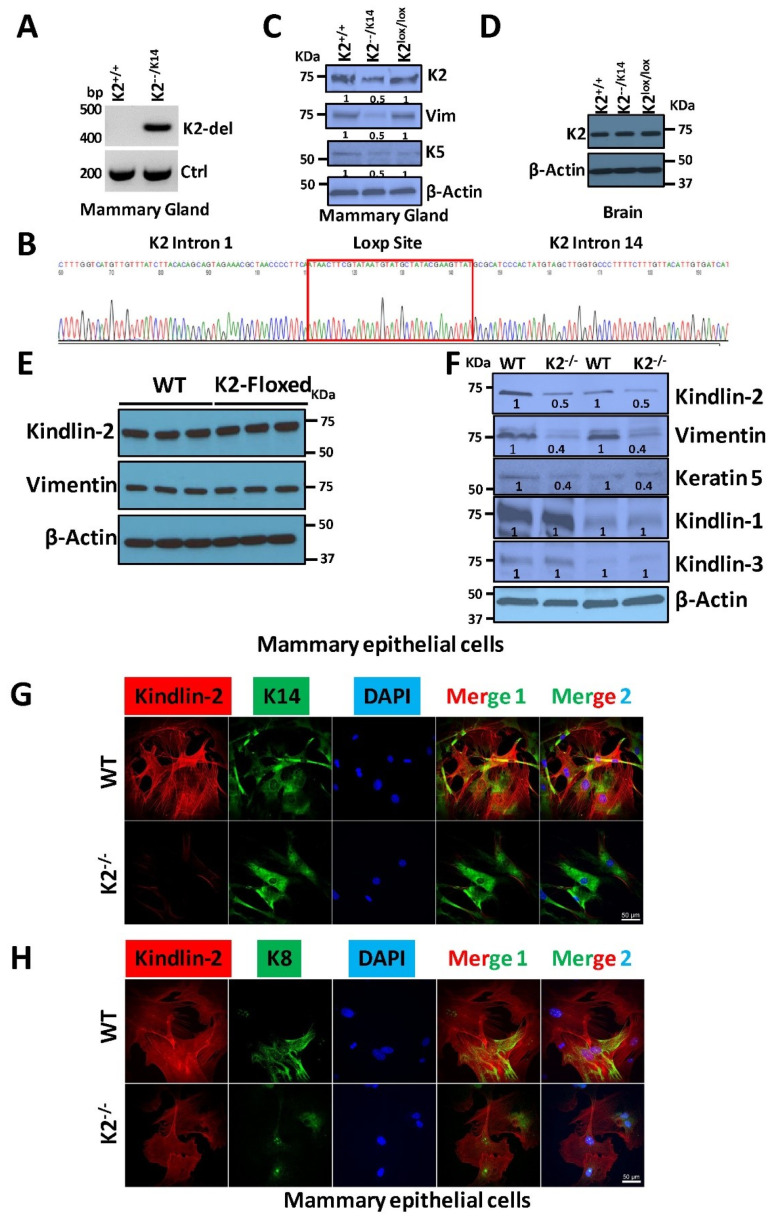
Tissue-specific deletion of *Fermt2* in mammary glands. (**A**) Representative agarose gel pictograms of the PCR products of DNA isolated from the mammary glands of wild-type (K2^+/+^) and K2-deficient mice (K2^−−/K14^) after crossing of the K2-Floxed mice with the K14-Cre mice. Only the K2^−−/K14^ mice show the ~404 bp PCR product, using the K2-del F&R primers, was amplified after deletion of the genomic sequence between Exon 1 and 14 (Figure 1A). (**B**) Sanger sequencing of the ~404 bp PCR product confirms the K2 intron 1 and intron 14 junction, separated by the Loxp site. (**C**,**D**) Representative Western blots of protein lysates probed with the indicated antibodies show loss of expression of Kindlin-2 (K2), Vimentin (Vim), and Keratin 5 (K5) in the mammary glands of K2^−−/K14^ mice, but not in those of wildtype and K2-floxed mice (**C**), while no change in K2 expression in all three mouse stains was observed when brain lysates were assessed (**D**). (**E**,**F**) Representative Western blots of protein lysates of mammary epithelial cells isolated from mammary glands of three different wild-type (WT) and three different K2^lox/lox^ (K2-Floxed) mice (**E**) and from mammary glands of two different wild-type (WT) and two different K2^−−/K14^ (K2^−/−^) mice (**F**), and probed with antibodies as indicated. β-Actin was used as loading control. The numbers under the WB bands represent the fold reduction of the signal with respect to the WT band after normalization to the β-Actin signal. Detailed information about the Western blotting can be found in Figure S3. (**G**,**H**) Representative confocal microscopy images of immunofluorescence staining of MECs isolated from mammary glands of wild-type or K2-deficient (K2^−/−^) mice. In Figure 2G, MECs were co-stained with anti-Kindlin-2 (red) or anti K14 (green) antibodies; in Figure 2H, MECs were co-stained with anti-Kindlin-2 (red) and anti-K8 (green) antibodies. DAPI (blue) was used to stain nuclei. Scale bar, 50 µm.

To generate mice with targeted deletion of Kindlin-2 in mammary epithelial cells, the K2^lox/lox^ mice were crossed with K14-Cre mice (B6N.Cg-Tg(KRT14-cre)1Amc/J mouse strain) from The Jackson Laboratory (Stock #018964), where Cre recombinase is expressed in the basal cell lineage of the mammary epithelium. Our choice of the K14-Cre mice to target the basal mammary epithelial cell lineage stems from data supporting the association of Kindlin-2 with the basal subtype of breast cancer [23,24,25]. Genomic PCR genotyping (Figure 2A) and sequencing (Figure 2B) confirmed the successful deletion of the genomic DNA sequence between intron 1 and intron 14 of the *Fermt2* gene from DNA extracted from mammary glands of K2^−−/K14^ mice. In the genotyping PCR reaction, a 404-bp band would only be amplified when the Cre recombination takes place between the 5′ and 3′ LoxP sites (Figure 1A). When this 404 bp PCR product was purified and sequenced, “fusion” of intron 1 with intron 14 was clearly seen as a result of the Cre recombinase-mediated deletion of the entire genomic sequence between intron 1 and intron 14 of the *Fermt2* gene (Figure 2B). Next, we used Western Blot analyses to assess expression levels of Kindlin-2 in the mammary glands of wild-type K2^lox/lox^ and K2^−−/K14^ mice and found a ~50% decrease in Kindlin-2 expression levels in mammary glands of K2^−−/K14^ mice, compared with the wild-type mice, while no changes in Kindlin-2 levels were observed in the mammary glands of K2^lox/lox^ mice (Figure 2C). On the other hand, no changes in Kindlin-2 expression levels were observed in the brains of all three mouse strains, where K14-Cre would have no effect on the *Fermt2* gene expression (Figure 2D). Specific deletion of the *Fermt2* gene and the subsequent loss of expression of Kindlin-2 in the mammary glands was further confirmed in mammary epithelial cells isolated from mammary glands of wild-type, K2-floxed, or K2^−/−^ mice (Figure 2E,F). First, we observed no changes in expression levels of Kindlin-2 in the mammary epithelial cells of wild-type and K2-floxed mice (Figure 2E). This observation demonstrates that insertion of the loxP sites in the Fermt2 locus had no effect on the expression of Kindlin-2. Of note, our published studies have shown that Kindlin-2 is involved in the regulation of the epithelial to mesenchymal transition (EMT) process [24]. Accordingly, here, we showed that, in the purified mammary epithelial cells of K2^−/−^ mice, in addition to loss of expression of Kindlin-2, both Vimentin and K5 Keratin, two EMT markers, were also reduced by ~50% in mammary glands of K2^−/−^ mice (Figure 2F). We further confirmed the loss of Kindlin-2 in the K2^−/−^ MECs using RT-PCR of cDNA generated from mRNA isolated from either MECS of K2^+/+^ or K2^−/−^ mice, and a primer pair flanking exon 1 (K2-EX1F) and exon 15 (K2-Ex15R) to generate a ~500 bp RT-PCR product only in the K2^−/−^ MECs (Figure S1A). A PCR product could not be detected in the WT-MECs since the length of the Kindlin-2 cDNA sequence to be amplified in the WT-MECs was more than 2200 bp, which was beyond the sensitivity of our RT-PCR setting. Sequencing of the ~500 bp RT-PCR product from the K2^−/−^ MECs (Figure S1B) showed a clear fusion junction between Exon 1 and Exon 15 of the Kindlin-2 transcript, which resulted in a premature stop codon after a continuous reading of seven additional amino acids in Exon 15 (Figure S1C). The resulting translated protein was 59-amino acid long and had a predicted molecular weight of ~7 kDa. We also used immunofluorescence analyses to confirm that Kindlin-2 was specifically deleted in the basal (K14-Cre driven deletion) mammary epithelial lineage (Figure 2G,H). In mammary epithelial cells isolated from wildtype mice, Kindlin-2 could be found in cells expressing the basal marker (K14) (Figure 2G, upper panel) as well as the cells expressing the luminal marker (K8) (Figure 2H, upper panel). However, in the mammary epithelial cells isolated from the mammary glands of K2^−/−^ mice, Kindlin-2 could only be found in cells expressing K8 (Figure 2H; lower panel), but not K14-expressing MECs (Figure 2G, lower panel). Together, these findings confirmed the loss of expression of Kindlin-2 in the basal cell population in the mammary glands.

### 3.3. Loss of Kindlin-2 in Basal Mammary Epithelial Cells Has No Deleterious Effects on Mammary Gland Development, Mouse Development and Fertility

**Figure 3:**
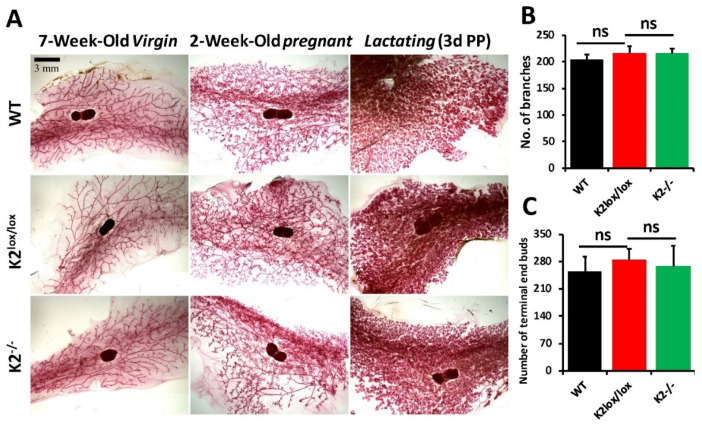
Loss of Kindlin-2 in the basal mammary epithelial cells has no deleterious effects on mammary gland development. (**A**) Whole mounts of mammary glands from wild-type (WT), K2-floxed (K2^lox/lox^), and K2-deficient mice (K2^−/−^) at the indicated gestational stages. Scale bar, 3 mm. (**B**) Quantification of the mammary gland branches from the 7-week-old virgin mice. (**C**) Quantification of the terminal end buds of mammary glands from the 2-week-old pregnant mice. ImageJ software was used for quantifications. Data are the means ± SD (*n* = 3, ns, not significant; Student’s *t*-test).

When the Kindlin-2^lox/lox^ mice were crossed with the K14-Cre mice, they produced litters of similar size and number compared to those obtained with wild-type mice, and did not show any signs of defects in development and fertility. Since our study is focused on the role of Kindlin-2 in breast cancer development, we focused our evaluation on mammary glands and assessed whole mounts of mammary glands at different stages of development and gestation (Figure 3A). We did not observe any obvious phenotypic differences between mammary glands of wild-type, K2^lox/lox^, and K2^−/−^ in 7-week-old virgin and 2-week-old pregnant and lactating mice. We also did not find any significant differences in the number of mammary gland branches (Figure 3B) and terminal-end buds (Figure 3C). Thus, our data suggested that neither floxing the Kindlin-2 locus nor deletion of Kindlin-2 in the mammary epithelial cells has any overt effect on normal mammary gland development and function.

### 3.4. Loss of Kindlin-2 Inhibits the Oncogenic Behavior of E0771 TNBC Cells In Vitro and In Vivo

**Figure 4:**
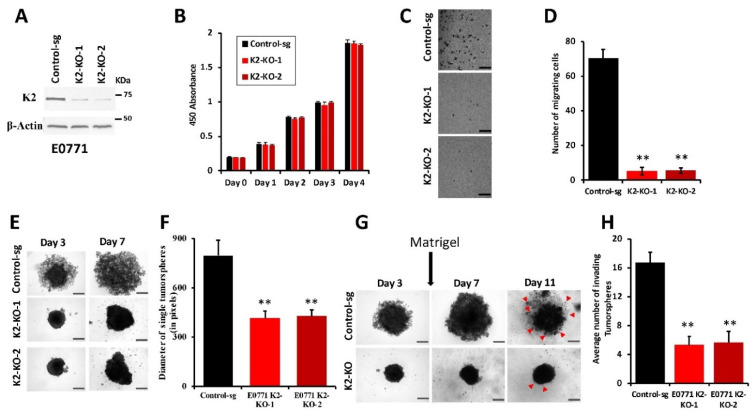
Loss of Kindlin-2 inhibits the oncogenic behavior of E0771 TNBC cells in vitro. (**A**) Representative Western blots of protein lysates developed with anti-Kindlin-2 antibody from E0771 cells transduced with a scrambled sgRNA (Control-sg), Kindlin-2 sgRNA-1 (K2-KO-1), or Kindlin-2 sgRNA-1 (K2-KO-2). β-Actin is a loading control. (**B**) Cell proliferation over 4 days. Data are the means ± SD (*n* = 3, ns, not significant; Student’s *t*-test). (**C**) Representative micrographs of parental E0771 and its K2-KO derivatives induced to form tumorspheres in the multiple-sphere formation assay. Scale bar, 40 µm. (**D**) Quantification of the number of tumorspheres at day 7. (**E**) Representative micrographs of parental E0771 and its K2-KO derivatives induced to form tumorspheres in the single-sphere formation assay. Scale bar, 200 µm. (**F**) Quantification of the diameter occupied by the resulting tumorspheres at day 7. (**G**) Representative micrographs of invading tumorspheres from parental E0771 and its K2-KO derivatives. Single spheres were grown in 96-well ultra-low adhesion plates and Matrigel (2.5 *v*/*v*) was added at day 3 and images were captured at day 7 and 11. Red arrows represent invading microspheres. Scale bar, 150 µm. (**H**) Quantification of the number of invading microspheres at day 11. Data are the means ± SD (*n* = 3, **, *p* < 0.001; Student’s *t*-test). Detailed information about the Western Blotting can be found in Figure S4.

In this study, we used the C57BL/6J mouse strain to generate Kindlin-2-deficient mice in mammary epithelial cells with the ultimate goal of assessing the consequences of mammary gland-specific knockout of Kindlin-2 on breast cancer development and metastasis. To undertake these analyses, a syngeneic breast cancer cell line model that is compatible with the C57BL/6J genetic background was needed. The only model that is currently available is the E0771 cell line, which also exhibits characteristics of triple-negative BC cells [32,33]. When injected into the mammary fat pads of C57BL/6j mice, E0771 cells rapidly establish very aggressive tumors that metastasize to the lungs and liver as early as two weeks after injection [26]. Likewise, E0771 cells are also highly proficient in colonizing the lungs of mice following their inoculation into the lateral tail vein [26]. The prominent role of Kindlin-2 in migration and invasion of cancer cells of multiple origins including breast cancer cells [24,25] is well established. In view of the value of E0771 cells as a syngeneic model of TNBC, we sought to determine whether Kindlin-2 plays an important role in the oncogenic activities of E0771 BC cells. Accordingly, we established E0771 derivatives with stable knockout of Kindlin-2 using CRISPR/Cas9-mediated gene editing (Figure 4A). Two different sgRNAs targeting two different exons of *Fermt2* [23,24,25] were used to establish two independent pools of Kindlin-2-knockout cell population (K2-KO-1 and K2-KO-2), each displaying >90% decrease in Kindlin-2 expression levels (Figure 4A). A scrambled sgRNA (Control-sg), i.e., nonsilencing sgRNA, [23,24,25], was used a control. First, it was important to evaluate whether loss of Kindlin-2 expression affected cell proliferation. No effect was seen over a four-day period in comparing the two K2-KO pools to the parental E0771 cells (Figure 4B). We used 3-dimensional (3D) tumorsphere growth and invasion assays to assess the role of loss of Kindlin-2 in E0771 cell growth and invasion in 3D conditions. We found loss of Kindlin-2 (K2-KO) in E0771 cells significantly (*p* < 0.001) inhibited the number (Figure 4C,D) and size (Figure 4E,F) of resulting tumorspheres as compared with the parental cells. Additionally, we found loss of Kindlin-2 expression significantly (*p* < 0.001) inhibited Matrigel invasion of E0771 cells (Figure 4G,H). Therefore, our findings establish the involvement of Kindlin-2 in specific oncogenic behaviors of E0771 breast cancer cells in vitro.

**Figure 5:**
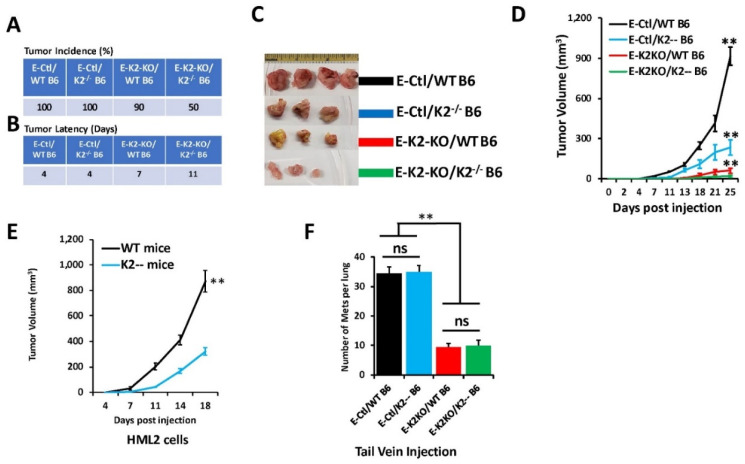
Loss of Kindlin-2 in the mammary glands inhibits the oncogenic behavior of E0771 TNBC cells in vivo. (**A**,**B**) Tumor incidence (**A**) and latency in days (**B**) of the animal experiment as described. (**C**) Pictures of tumors and their comparative sizes from the different groups from the animal experiment as described. (**D**) Quantification of volume of tumors generated from inoculation of control E0771 cells or their K2-KO derivatives into the mammary fat pads of female C57BL/6J mice or their K2^−/−^ derivatives. (**E**) Quantification of tumor volume generated from inoculation of control HML2 cells into the mammary fat pads of female wild-type C57BL/6J or K2-deficient (K2^−/−^) mice. (**F**) Quantification of lung metastasis after tail vein injection of control E0771 cells or their K2-KO derivatives of female wild-type C57BL/6J (WT) or K2-deficient (K2^−/−^) mice. Data are generated from five mice per group. Data are the mean ± SD (*n* = 5, ** *p* < 0.001; Student’s *t*-test).

Next, we assessed the effects of loss of Kindlin-2 in E0771 cells on tumor growth in vivo. Mammary fat pads of wildtype (WT-B6) or K2^−/−^ (K2^−/−^ B6) C57BL/6 mice were inoculated with either parental (E-Ctl) or K2-KO (E-K2-KO) E0771 derivatives, and tumor growth was assessed over 4 weeks. Every mouse in the control (E-Ctl) group developed tumors in all injection sites, and in both the wild-type (WT B6) and the K2-deficient K2^−/−^ B6) mice (100% tumor incidence; Figure 5A,C). A ∼4-day latency was observed before tumors developed in both groups (Figure 5B,C). Tumor incidence in the E0771-K2KO group (E-K2-KO), on the other hand, remained high (90%), although tumor latency extended from a period of 7 days in wild-type mice (WT B6) to 11 days in K2^−/−^ mice (Figure 5A,B). Tumor burden (tumor volume) was also significantly lower (*p* < 0.001) in wild-type mice implanted with the Kindlin-2-deficient cells as compared with their control counterparts (Figure 5D, compare black graph to red graph). The same observation was noted for the K2^−/−^ mice that developed smaller tumors (*p* < 0.001) when injected with K2-KO E0771 cells when compared with the same mice injected with the parental E0771 controls (Figure 5D, compare blue graph to green graph). Thus, loss of Kindlin-2 inhibited the rate of primary tumor growth in vivo in our E0771 TNBC murine syngeneic model. When assessing the effect of loss of Kindlin-2 in mammary glands on tumor growth of E0771 cells, a significant (*p* < 0.001) reduction in tumor growth (Figure 5D, compare black graph to blue graph) was found when parental E0771 (E-Ctl) cells were injected in the mammary fat pads of K2-deficient (K2--B6) mice as compared to wild-type (WT B6) mice injected with the control E0771 cells. We also found a loss of Kindlin-2 in E0771 cells inhibited tumor growth when injected in the mammary fat pads of mice with K2-deficient mammary glands compared with wild-type mice (Figure 5D, compare red graph to green graph). Thus, double knockout of Kindlin-2 in both the cancer cells and the mammary glands has a more profound inhibitory effect on tumor growth than Kindlin-2 knockout in either cancer cells or mammary glands, separately, further supporting the regulatory role of Kindlin-2 in both cancer cells and in the tumor microenvironment. These findings were replicated with the more aggressive HML2 cells (Figure 5E). The HML2 cells were derived from E0771 through serial passaging in mice and have higher metastatic potential than the parental E0771 cells [26]. The K2-deficient (K2^−/−^) mice injected with HML2 cells developed significantly smaller tumors (*p* < 0.001) as compared with wild-type mice (Figure 5D), thereby reinforcing the oncogenic activities of K2 in a second syngeneic mouse model of TNBC. Even though HML2 cells were derived from the E0771 cells, and do not represent a true independent replicate, they still provide a second confirmatory cell model for our tumor growth assay. To further confirm the role of mammary gland Kindlin-2 in the regulation of the growth of the primary breast cancer tumors, we bypassed the primary tumor site (mammary glands) and injected the E0771 cells or their K2-deficient derivatives directly into the tail veins of either wild-type or K2^−/−^ mice (Figure 5F). We found the lung metastasis burden was significantly (*p* < 0.001) higher in the mice injected with the parental E0771 (E-Ctl) cells as compared with their K2-KO derivatives, regardless of K2 status in the mammary gland (Figure 5F, compare black and blue bars to red and green bars). However, we found no significant difference in lung metastasis colonization between the K2-deficient and the wild-type mice when injected with E0771 cells with similar K2 genetic status (Figure 5F, compare black graph to blue graph for parental E0771 cells, and red graph to green graph to K2-KO E0771 cells). These data further support the notion that loss of Kindlin-2 in the mammary glands contributes to the inhibition of tumor growth in the primary site, and support a critical role of Kindlin-2, not only in the cancer cells but also in the epithelial cells of the mammary gland in tumor development.

### 3.5. Kindlin-2 Regulates the TGF-β/EGF Signaling Axis in MECs to Support Breast Cancer Tumor Growth in Mammary Glands

**Figure 6:**
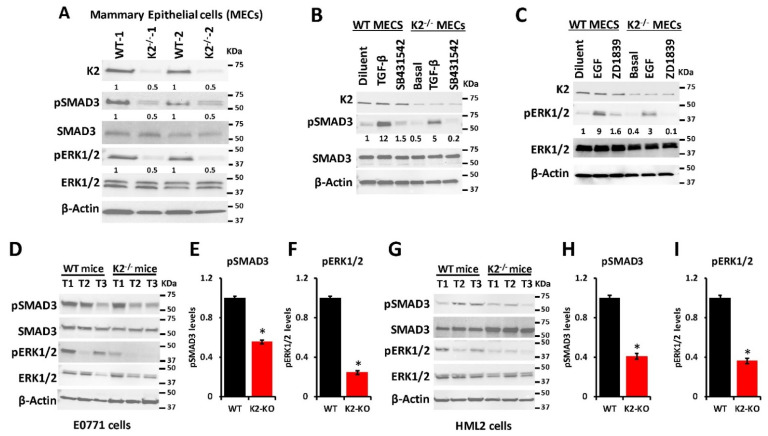
Kindlin-2 regulates the TGF-β/EGF signaling axis in MECs to support breast cancer tumor growth in mammary glands. (**A**) Representative Western blots of protein lysates of mammary epithelial cells isolated from mammary glands of two different wild-type (WT) and two different K2^−/−/K14^ (K2^−/−^) mice, probed with antibodies against the indicated phospho proteins and their total counterparts. β-Actin is a loading control. (**B**,**C**) WT or K2-deficient (K2^−/−^) MECs were treated as indicated and subjected to immunoblotting with antibodies against the indicated phospho proteins and their total counterparts. β-Actin is a loading control. (**D**–**I**) Western Blot analyses with the indicated antibodies of protein lysates from tumors (T1–T3) derived from either E0771 (**D**) or HML2 (**G**) that were injected in the mammary fat pad of wild-type mice or their K2^−/−^ derivatives. β-Actin is a loading control. Signal quantification for pSMAD3 and pERK1/2 are shown in (**E**,**F**) for the E0771-derived tumors, and in (**H**,**I**) for the HML2-derived tumors. The numbers under the WB bands represent the fold reduction of the signal with respect to the WT or the diluent band after normalization to the β-Actin signal. Data are representative of three independent replicates. Data are the mean ± SD (* *p* < 0.01; Student’s *t*-test). Detailed information about the Western blotting can be found in Figures S5–S7.

Our previously published study [25] showed that Kindlin-2 in tumor cells was involved in the regulation of the CSF1/EGF oncogenic loop, downstream of TGF-β, to activate growth and metastasis of TNBC tumors in mice. The extent to which Kindlin-2 within mammary epithelial cells is also involved in the regulation of the oncogenic TGF-β/EGF signaling axis is, however, not known. Accordingly, we used Western Blot analysis to show that loss of Kindlin-2 in the K2^−/−^ MECs resulted in a significant (*p* < 0.01) reduction of pSMAD3 and pERK1/2 as downstream effectors of TGF-β and EGF, respectively (Figure 6A). These results mirror those obtained in the MDA-MB-231 and 4T1 TNBC cells [25]. To further confirm Kindlin-2 involvement in mammary epithelial cells to regulate tumor growth, we isolated mammary epithelial cells from wild-type or K2-deficient mice and treated them with either TGF-β (Figure 6B) or EGF (Figure 6C), and subsequently measured their activation of either SMAD3 (Figure 6B) or ERK1/2 (Figure 6C), respectively. In the TGF-β-treated MECs, pSMAD3 levels were 2.4-fold lower in the K2-deficient MECS as compared with their wild-type counterparts (Figure 6C). Similarly, pERK levels were 3-fold lower in the K2-deficient MECs after treatment with EGF as compared with their wild-type counterparts (Figure 6C). Treatment with TGF-β or EGF signaling inhibitors (SB4131542 and ZD1839, respectively) blocked the activation of their respective downstream effectors (Figure 6B,C), further supporting the Kindlin-2-mediated regulation of the TGF-β/EGF signaling axis role in mammary glands. We also found both EGF and TGFβ signaling to be significantly lower in the tumors derived from E0771 (Figure 6D–F) and HML2 (Figure 6G–I) when injected in K2^−/−^ mice as compared with their wild-type counterparts, further supporting our in vitro findings with in vivo data. Together, our data showed that Kindlin-2, in both the tumor cells and the tumor microenvironment (MECs), is required for the regulation of the TGF-β/EGF signaling axis and subsequent activation of tumor growth.

## 4. Discussion

Several independent studies, including those from our group, have demonstrated the critical role Kindlin-2 plays in cancer progression and metastasis (reviewed in [20,21,22]). These studies involved manipulation of Kindlin-2 expression levels using siRNA, shRNA, and CRISPR/Cas9 gene editing, thereby assessing its oncogenic activities both in vitro analyses and in vivo [23,24,25,34,35]. The in vitro studies have proven to be very useful in identifying, characterizing, and evaluating molecular mechanisms whereby Kindlin-2 is involved in regulating cancer cell proliferation, migration, and invasion. These in vitro studies were supported by mouse models of tumor progression and metastasis in xenograft studies using the genetically manipulated cells. Together, these studies have identified Kindlin-2 as a major player in the regulation of the invasion-metastasis cascade, both in the cancer cells as well as in the tumor microenvironment [13,25]. It should be noted, however, that these studies consistently showed that loss of Kindlin-2 function does not lead to total elimination of invasion and metastasis, possibly because both shRNA knockdown and CRISPR/Cas9-mediated knockout used in these studies did not result in complete loss of Kindlin-2 expression in the cancer cells. Therefore, we posited that the residual K2 expression in cancer cells may have been responsible for not eliminating metastasis completely. Accordingly, we sought to investigate the effect of loss of Kindlin-2 expression in mammary glands development and malignant transformation. Since homozygous deletion of mouse *Fermt2* results in embryonic lethality, we implemented a floxed mouse strategy and crossed these mice with a tissue-specific K14-Cre mouse strain, where Cre recombinase is preferably expressed in tissues and cells with high levels of K14 Keratin, which is also highly expressed in the basal subtype of mammary epithelial cells.

Loss of expression of Kindlin-2 in the basal subtype of mammary epithelial cells did not have any appreciable effects on normal mammary gland development and ductal expansion during distinct stages of development, e.g., virgin, pregnancy, and post-partum (Figure 3). Fertility was also not affected, since pups were delivered at the expected ratio and exhibited a normal developmental pattern as compared with their WT counterparts. Loss of Kindlin-2 in the mammary glands did, however, have a significant inhibitory effect on tumor growth and metastasis. This inhibitory effect may be specific to the loss of expression of Kindlin-2 in the mammary glands since injecting tumor cells via tail veins and thereby bypassing critical steps of the invasion-metastasis cascade failed to impact the overall burden of lung metastasis between the WT mice and their Kindlin-2-deficient counterparts. It is, however, very important to note that K14 is also expressed in the lungs as well as in other tissues such as the esophagus, tongue, thymus, and even the skin. Indeed, our genotyping analyses showed that crossing the K2 floxed mice with the Cre-K14 mice resulted in targeting K2 in these tissues as well, but this targeting was very minimal and had no effect on the normal development of mice bearing the transgenic K2 deletion. On the other hand, since K14 is also expressed in the lungs, this possibly may have influenced lung metastasis in the K2-targeting using the K14-Cre mice. However, our data in Figure 5 showed no difference in lung metastasis between WT and K2-deficient mice when injected with the same cells (parental or K2-KO E0771 cells).

While our syngeneic orthotopic model showed Kindlin-2 in MECs was critical in BC cancer progression and metastasis, it remains to be seen whether this effect would be maintained in mouse models that develop BC spontaneously, such as the MMTV-PyMT [36], MMTV-Myc [37] and MMTV-Wnt1 [38] models. Our long-term investigations will test the effect of loss of Kindlin-2 on tumor growth and metastasis in such spontaneous mouse models.

TGF-β signaling plays both anti- and pro-tumorigenic roles in breast cancer as well as in other tumor types [39,40]. In physiological conditions, TGF-β is essential in maintaining tissue homeostasis by inducing cell cycle arrest, differentiation, apoptosis, and preserving genomic stability, functioning as an anticancer agent that inhibits uncontrolled cell proliferation. This anti-tumor activity of TGF-β can still be observed in early stages of cancer [39,40]. However, in advanced stages of tumor development, the cytostatic function of TGF-β transits from a suppressor of tumor formation to a promoter of tumor growth, invasion, and metastasis, referred to as the TGF-β Paradox [40,41]. Several studies have now demonstrated an interrelationship between Kindlin-2 and TGF-β in physiological conditions [13,42,43,44] as well as in pathological settings, including tumor progression and metastasis ([24,25,45] and renal fibrosis [46]). Our study [25] identified a Kindlin-2/TGF-β/CSF-1 signaling axis, whereby TGF-β signaling modulates cancer cell Kindlin-2-mediated regulation of a CSF-1/EGF autocrine and paracrine oncogenic loop that regulates the crosstalk between cancer cells and type 2 tumorigenic macrophages to promote growth and metastasis of BC tumors. Surprisingly, our findings seem to involve Kindlin-2 in mammary epithelial cells in a similar Kindlin-2/TGF-β/EGF signaling axis, where loss of Kindlin-2 in MECs also inhibited the downstream effectors of both TGF-β (pSMAD2/3) and EGF (pERK1/2). Stimulation of the K2-deficient mammary epithelial cells with either TGF-β or EGF was not able to restore their respective activities to levels seen in the wild-type MECs (Figure 6). Thus, our data support the role of Kindlin-2 in both cancer cells (4T1, MDA-MB-231 [24,25], and E0771 and HML2 BC cells, present study) and the tumor microenvironment (MECs), which suggests that a potential successful treatment of BC may require a dual targeting of Kindlin-2 and/or its downstream signaling in both the tumor and the tumor microenvironment. Notably, targeting of K2 in mammary epithelial cells did not affect mammary gland development or function.

The present study showed the utility of the floxed Kindlin-2 mice to demonstrate the role of Kindlin-2 expressed in mammary epithelial cells in BC progression and metastasis. Because of the involvement of Kindlin-2 in the pathogenicity of other cancers, this new reagent may prove to be very useful in future investigations. In fact, our published studies have shown that Kindlin-2 regulates sensitivity to chemotherapeutics in prostate cancer [34]. The floxed Fermt2 strain, therefore, provides a unique opportunity to investigate the role of Kindlin-2 in other cancer models such as the transgenic adenocarcinoma of the mouse prostate (TRAMP) model for prostate cancer. Our ongoing studies (unpublished data) have already used this mouse strain to investigate the role of Kindlin-2 in vascular cell types.

## Figures and Tables

**Table 1:** List and sequences of oligonucleotide primers.

Primer Name	Primer Sequence	PCR Product Size (bp)
K2 Floxed 5′ F	5′-CTT CCC TCA GTG ATG GAG TGT GAT CTG AG-3′	230/264
K2 Floxed 5′ R	5′-GGA GTC AGA GAG AAT GGG CAC TCT AGG TG-3′	
K2 Floxed 3′ F	5′-CTA AAG CAG GCA GGT TGC CTG GAC-3′	288/322
K2 Floxed 3′ R	5′-CTC TTA CCC ACT GAG CCA TCT CAC C-3′	
K2 Del F	5′-CCC TCA GTG ATG GAG TGT GAT-3′	404
K2 Del R	5′-AAG AGG GCG TCA GAT TTC GTT-3′	
K14 Cre Tg F	5′-GCG GTC TGG CAG TAA AAA CTA TC-3′	100
K14 Cre Tg R	5′-GTG AAA CAG CAT TGC TGT CAC TT-3′	
K2 RT Ex1 F	5′-CGG GAC TCC ATT AGC AGC G-3′	2201/457
K2 RT Ex 15 R	5′-TTC CTA TTC ACA CCC AAC CAC T-3′	

## Data Availability

The data presented in this study are available in this article (and Supplementary Material).

## Supplementary Materials

The following are available online at https://www.mdpi.com/article/10.3390/cancers14030639/s1, Figure S1: Cloning and sequencing of the Exon1-Exon15 RT-PCR product, Figure S2: Uncropped full blot for Figure 1, Figure S3: Uncropped full Western blot for Figure 2, Figure S4: Uncropped full Western blot for Figure 4A, Figure S5: Uncropped full Western blot for Figure 6A, Figure S6: Uncropped full Western blot for Figure 6B,C, Figure S7: Uncropped full Western blot for Figure 6D,G, File S1: Detailed description of the procedures used to generate the Kindlin-2.

## References

[1] Liu J., Fukuda K., Xu Z., Ma Y.Q., Hirbawi J., Mao X., Wu C., Plow E.F., Qin J. (2011). Structural basis of phosphoinositide binding to kindlin-2 protein pleckstrin homology domain in regulating integrin activation. J. Biol. Chem..

[2] Liu Y., Zhu Y., Ye S., Zhang R. (2012). Crystal structure of kindlin-2 PH domain reveals a conformational transition for its membrane anchoring and regulation of integrin activation. Protein Cell.

[3] Perera H.D., Ma Y.Q., Yang J., Hirbawi J., Plow E.F., Qin J. (2011). Membrane binding of the N-terminal ubiquitin-like domain of kindlin-2 is crucial for its regulation of integrin activation. Structure.

[4] Metcalf D.G., Moore D.T., Wu Y., Kielec J.M., Molnar K., Valentine K.G., Wand A.J., Bennett J.S., DeGrado W.F. (2010). NMR analysis of the alphaIIb beta3 cytoplasmic interaction suggests a mechanism for integrin regulation. Proc. Natl. Acad. Sci. USA.

[5] Larjava H., Plow E.F., Wu C. (2008). Kindlins: Essential regulators of integrin signalling and cell-matrix adhesion. EMBO Rep..

[6] Moser M., Nieswandt B., Ussar S., Pozgajova M., Fassler R. (2008). Kindlin-3 is essential for integrin activation and platelet aggregation. Nat. Med..

[7] Malinin N.L., Plow E.F., Byzova T.V. (2010). Kindlins in FERM adhesion. Blood.

[8] Ussar S., Moser M., Widmaier M., Rognoni E., Harrer C., Genzel-Boroviczeny O., Fassler R. (2008). Loss of Kindlin-1 causes skin atrophy and lethal neonatal intestinal epithelial dysfunction. PLoS Genet..

[9] Moser M., Bauer M., Schmid S., Ruppert R., Schmidt S., Sixt M., Wang H.V., Sperandio M., Fassler R. (2009). Kindlin-3 is required for beta2 integrin-mediated leukocyte adhesion to endothelial cells. Nat. Med..

[10] Dowling J.J., Gibbs E., Russell M., Goldman D., Minarcik J., Golden J.A., Feldman E.L. (2008). Kindlin-2 is an essential component of intercalated discs and is required for vertebrate cardiac structure and function. Circ. Res..

[11] Pluskota E., Dowling J.J., Gordon N., Golden J.A., Szpak D., West X.Z., Nestor C., Ma Y.Q., Bialkowska K., Byzova T. (2011). The integrin coactivator kindlin-2 plays a critical role in angiogenesis in mice and zebrafish. Blood.

[12] Ying J., Luan W., Lu L., Zhang S., Qi F. (2018). Knockdown of the KINDLIN-2 Gene and Reduced Expression of Kindlin-2 Affects Vascular Permeability in Angiogenesis in a Mouse Model of Wound Healing. Med. Sci. Monit..

[13] Wu C., Jiao H., Lai Y., Zheng W., Chen K., Qu H., Deng W., Song P., Zhu K., Cao H. (2015). Kindlin-2 controls TGF-beta signalling and Sox9 expression to regulate chondrogenesis. Nat. Commun..

[14] Zhang Z., Mu Y., Zhang J., Zhou Y., Cattaneo P., Veevers J., Peter A.K., Manso A.M., Knowlton K.U., Zhou X. (2019). Kindlin-2 Is Essential for Preserving Integrity of the Developing Heart and Preventing Ventricular Rupture. Circulation.

[15] Pluskota E., Ma Y., Bledzka K.M., Bialkowska K., Soloviev D.A., Szpak D., Podrez E.A., Fox P.L., Hazen S.L., Dowling J.J. (2013). Kindlin-2 regulates hemostasis by controlling endothelial cell-surface expression of ADP/AMP catabolic enzymes via a clathrin-dependent mechanism. Blood.

[16] Pluskota E., Bledzka K.M., Bialkowska K., Szpak D., Soloviev D.A., Jones S.V., Verbovetskiy D., Plow E.F. (2017). Kindlin-2 interacts with endothelial adherens junctions to support vascular barrier integrity. J. Physiol..

[17] Siegel D.H., Ashton G.H., Penagos H.G., Lee J.V., Feiler H.S., Wilhelmsen K.C., South A.P., Smith F.J., Prescott A.R., Wessagowit V. (2003). Loss of kindlin-1, a human homolog of the Caenorhabditis elegans actin-extracellular-matrix linker protein UNC-112, causes Kindler syndrome. Am. J. Hum. Genet..

[18] Mory A., Feigelson S.W., Yarali N., Kilic S.S., Bayhan G.I., Gershoni-Baruch R., Etzioni A., Alon R. (2008). Kindlin-3: A new gene involved in the pathogenesis of LAD-III. Blood.

[19] Malinin N.L., Zhang L., Choi J., Ciocea A., Razorenova O., Ma Y.Q., Podrez E.A., Tosi M., Lennon D.P., Caplan A.I. (2009). A point mutation in KINDLIN3 ablates activation of three integrin subfamilies in humans. Nat. Med..

[20] Plow E.F., Das M., Bialkowska K., Sossey-Alaoui K. (2016). Of Kindlins and Cancer. Discoveries (Craiova).

[21] Wang W., Kansakar U., Markovic V., Sossey-Alaoui K. (2020). Role of Kindlin-2 in cancer progression and metastasis. Ann. Transl. Med..

[22] Zhan J., Zhang H. (2018). Kindlins: Roles in development and cancer progression. Int. J. Biochem. Cell. Biol..

[23] Sossey-Alaoui K., Pluskota E., Szpak D., Plow E.F. (2019). The Kindlin2-p53-SerpinB2 signaling axis is required for cellular senescence in breast cancer. Cell Death Dis..

[24] Sossey-Alaoui K., Pluskota E., Szpak D., Schiemann W.P., Plow E.F. (2018). The Kindlin-2 regulation of epithelial-to-mesenchymal transition in breast cancer metastasis is mediated through miR-200b. Sci. Rep..

[25] Sossey-Alaoui K., Pluskota E., Bialkowska K., Szpak D., Parker Y., Morrison C.D., Lindner D.J., Schiemann W.P., Plow E.F. (2017). Kindlin-2 Regulates the Growth of Breast Cancer Tumors by Activating CSF-1-Mediated Macrophage Infiltration. Cancer Res..

[26] Kitamura T., Kato Y., Brownlie D., Soong D.Y.H., Sugano G., Kippen N., Li J., Doughty-Shenton D., Carragher N., Pollard J.W. (2019). Mammary Tumor Cells with High Metastatic Potential Are Hypersensitive to Macrophage-Derived HGF. Cancer Immunol. Res..

[27] Robinson G.W., McKnight R.A., Smith G.H., Hennighausen L. (1995). Mammary epithelial cells undergo secretory differentiation in cycling virgins but require pregnancy for the establishment of terminal differentiation. Development.

[28] Rana P.S., Kurokawa M., Model M.A. (2020). Evidence for macromolecular crowding as a direct apoptotic stimulus. J. Cell Sci..

[29] Bialkowska K., Sossey-Alaoui K., Pluskota E., Izem L., Qin J., Plow E.F. (2019). Site-specific phosphorylation regulates the functions of kindlin-3 in a variety of cells. Life Sci. Alliance.

[30] Kansakar U., Wang W., Markovic V., Sossey-Alaoui K. (2021). Phosphorylation of the proline-rich domain of WAVE3 drives its oncogenic activity in breast cancer. Sci. Rep..

[31] Wang W., Kansakar U., Markovic V., Wang B., Sossey-Alaoui K. (2020). WAVE3 phosphorylation regulates the interplay between PI3K, TGF-beta, and EGF signaling pathways in breast cancer. Oncogenesis.

[32] Casey A.E., Laster W.R., Ross G.L. (1951). Sustained enhanced growth of carcinoma EO771 in C57 black mice. Proc. Soc. Exp. Biol. Med..

[33] Johnstone C.N., Smith Y.E., Cao Y., Burrows A.D., Cross R.S., Ling X., Redvers R.P., Doherty J.P., Eckhardt B.L., Natoli A.L. (2015). Functional and molecular characterisation of EO771.LMB tumours, a new C57BL/6-mouse-derived model of spontaneously metastatic mammary cancer. Dis. Models Mech..

[34] Sossey-Alaoui K., Plow E.F. (2016). miR-138-Mediated Regulation of KINDLIN-2 Expression Modulates Sensitivity to Chemotherapeutics. Mol. Cancer Res..

[35] Bledzka K., Bialkowska K., Sossey-Alaoui K., Vaynberg J., Pluskota E., Qin J., Plow E.F. (2016). Kindlin-2 directly binds actin and regulates integrin outside-in signaling. J. Cell Biol..

[36] Guy C.T., Cardiff R.D., Muller W.J. (1992). Induction of mammary tumors by expression of polyomavirus middle T oncogene: A transgenic mouse model for metastatic disease. Mol. Cell Biol..

[37] Sinn E., Muller W., Pattengale P., Tepler I., Wallace R., Leder P. (1987). Coexpression of MMTV/v-Ha-ras and MMTV/c-myc genes in transgenic mice: Synergistic action of oncogenes in vivo. Cell.

[38] Tsukamoto A.S., Grosschedl R., Guzman R.C., Parslow T., Varmus H.E. (1988). Expression of the int-1 gene in transgenic mice is associated with mammary gland hyperplasia and adenocarcinomas in male and female mice. Cell.

[39] David C.J., Massague J. (2018). Contextual determinants of TGFbeta action in development, immunity and cancer. Nat. Rev. Mol. Cell Biol..

[40] Tian M., Schiemann W.P. (2009). The TGF-beta paradox in human cancer: An update. Future Oncol..

[41] Bierie B., Moses H.L. (2006). Tumour microenvironment: TGFbeta: The molecular Jekyll and Hyde of cancer. Nat. Rev. Cancer.

[42] Godbout E., Son D.O., Hume S., Boo S., Sarrazy V., Clement S., Kapus A., Wehrle-Haller B., Bruckner-Tuderman L., Has C. (2020). Kindlin-2 Mediates Mechanical Activation of Cardiac Myofibroblasts. Cells.

[43] Hirschberg R. (2013). Kindlin-2: A new player in renal fibrogenesis. J. Am. Soc. Nephrol..

[44] Qu H., Tu Y., Shi X., Larjava H., Saleem M.A., Shattil S.J., Fukuda K., Qin J., Kretzler M., Wu C. (2011). Kindlin-2 regulates podocyte adhesion and fibronectin matrix deposition through interactions with phosphoinositides and integrins. J. Cell Sci..

[45] Zhan J., Song J., Wang P., Chi X., Wang Y., Guo Y., Fang W., Zhang H. (2015). Kindlin-2 induced by TGF-beta signaling promotes pancreatic ductal adenocarcinoma progression through downregulation of transcriptional factor HOXB9. Cancer Lett..

[46] Wei X., Xia Y., Li F., Tang Y., Nie J., Liu Y., Zhou Z., Zhang H., Hou F.F. (2013). Kindlin-2 mediates activation of TGF-beta/Smad signaling and renal fibrosis. J. Am. Soc. Nephrol..

