# Supplementary material for: Targeted Deletion of Kindlin-2 in Mouse Mammary Glands Inhibits Tumor Growth, Invasion, and Metastasis Downstream of a TGF-β/EGF Oncogenic Signaling Pathway
Source: Cancers (Basel). 2022 Jan 27;14(3):639. doi: 10.3390/cancers14030639 (PMC8833458; doi:10.3390/cancers14030639)
Supplement: Supplementary file 1 [file cancers-14-00639-s001.zip › cancers-1567837-supp-final/cancers-1508892-File S1.pdf]

Supplementary Information.

File S1. Detailed description of the procedures used to generate the Kindlin-2 (Fermt2) floxed mouse strain by Applied StemCell.

Generation of F1 heterozygotes from F0 founders

Customer information

**Project Title**	**mFermt2-cKO**
**Job Ticket**	**MC096**
**Client Name**	**Ed Plow, Ph.D.**
**Client Institute**	**Cleveland Clinic**
**Report Date**	**02/18/2018**

Summary

To generate F1 heterozygotes, the F0 founders containing both upstream and downstream of loxp insertion at the targeted introns were set up for breeding with the wild type C57BL/6J. The F1 pups derived from the F0 founders were genotyped for selection of the heterozygotes with both upstream and downstream loxps presence at the targeted sites.

Genotyping analysis

Tail or ear tissues were collected from individual pups at age of d5 to 10 post birth. Genomic DNA was extracted from the tissue samples. PCR/NGS with genomic DNA as a template were applied to analyze the targeted sites. PCR/NGS analysis were applied to both 5' and 3' targeted-sites to screen for the pups with both 5' and 3' loxp insertion.

Table 1. Primers used in identification of Fermt2 mutants

Primer name	Primer sequence	Amplicon (bp)
**Genotyping PR 5F**	5'-CTTCCCTCAGTGATGGAGTGTGATCTGAG-3'	230/264
**Genotyping PR 3R**	5'-GGAGTCAGAGAGAATGGGCACTCTAGGTG-3'	
**Genotyping PR 3F**	5'-CTAAAGCAGGCAGGTTGCCTGGAC-3'	288/322
**Genotyping PR 3R**	5'-CTCTTACCCACTGAGCCATCTCACC-3'	

Results

Several founders including 3024#2, Male and 3024#3, female (DOB 10/16/17) were bred with wild type mice. From 3024#3 female F0 founder two litters with total 12 pups were born. After PCR/sequencing analysis, 5 pups (2 pups in the 1st litter, A litter were not saved due to wrong decision) were determined to be heterozygotes with perfect insertion of the loxps at both intron sites (NGS data for 3024#3B#2, 5, 8 from 2nd litter, B litter were shown below Fig1).

MC096-5LP-1-30243B-2

```
MC096-5LP-30243B-2_5742_63.3    GTAAGCTGAAATAAACCCTTTCTTCCTCAGTCACTTTGGTCATGTTGTTTATCTTACACA
MC096-5LP-30243B-2_3324_36.7    GTAAGCTGAAATAAACCCTTTCTTCCTCAGTCACTTTGGTCATGTTGTTTATCTTACACA
MC096-5LP_ref                   GTAAGCTGAAATAAACCCTTTCTTCCTCAGTCACTTTGGTCATGTTGTTTATCTTACACA

MC096-5LP-30243B-2_5742_63.3    GCAGTAGAAACGCTAACCCCTTCA----------------------------------GT
MC096-5LP-30243B-2_3324_36.7    GCAGTAGAAACGCTAACCCCTTCAATAACTTCGTATAATGTATGCTATACGAAGTTATGT
MC096-5LP_ref                   GCAGTAGAAACGCTAACCCCTTCAATAACTTCGTATAATGTATGCTATACGAAGTTATGT

MC096-5LP-30243B-2_5742_63.3    TACCGTCCTCAGGATATATTTTGGAAGCATGTTTGGGCTGCAAGCTGTTCATGGTTGTTC
MC096-5LP-30243B-2_3324_36.7    TACCGTCCTCAGGATATATTTTGGAAGCATGTTTGGGCTGCAAGCTGTTCATGGTTGTTC
MC096-5LP_ref                   TACCGTCCTCAGGATATATTTTGGAAGCATGTTTGGGCTGCAAGCTGTTCATGGTTGTTC

MC096-5LP-30243B-2_5742_63.3    TGGAGTTCTTTTCACGCCCCT
MC096-5LP-30243B-2_3324_36.7    TGGAGTTCTTTTCACGCCCCT
MC096-5LP_ref                   TGGAGTTCTTTTCACGCCCC-
```

MC096-3LP-1-30243B-2

```
MC096-3LP-30243B-2_844_61.3    GCTCCCACTTAGAAGCCTAAGGGCTGCTTAGTGTCCACGCCCACACACTTGATGACTGCC
MC096-3LP-30243B-2_533_38.7    GCTCCCACTTAGAAGCCTAAGGGCTGCTTAGTGTCCACGCCCACACACTTGATGACTGCC
MC096-3LP_ref                  GCTCCCACTTAGAAGCCTAAGGGCTGCTTAGTGTCCACGCCCACACACTTGATGACTGCC

MC096-3LP-30243B-2_844_61.3    GCAGCAGGGTCTGTAAAACCTGG----------------------------------TGC
MC096-3LP-30243B-2_533_38.7    GCAGCAGGGTCTGTAAAACCTGGTATAACTTCGTATAATGTATGCTATACGAAGTTATGC
MC096-3LP_ref                  GCAGCAGGGTCTGTAAAACCTGGTATAACTTCGTATAATGTATGCTATACGAAGTTATGC

MC096-3LP-30243B-2_844_61.3    GCATCCCACTATGTAGCTTGGTGCCCTTTTCTTTGTTACATTGTGATCATATAAAACCGG
MC096-3LP-30243B-2_533_38.7    GCATCCCACTATGTAGCTTGGTGCCCTTTTCTTTGTTACATTGTGATCATATAAAACCGG
MC096-3LP_ref                  GCATCCCACTATGTAGCTTGGTGCCCTTTTCTTTGTTACATTGTGATCATATAAAACCGG

MC096-3LP-30243B-2_844_61.3    CTCACTGGCAGGCGATTTTG
MC096-3LP-30243B-2_533_38.7    CTCACTGGCAGGCGATTTTG
MC096-3LP_ref                  CTCACTGGCAGGCGATTTTG
```

MC096-5LP-1-30243B-5

```
MC096-5LP-30243B-5_5639_63.1    GTAAGCTGAAATAAACCCTTTCTTCCTCAGTCACTTTGGTCATGTTGTTTATCTTACACA
MC096-5LP-30243B-5_3302_36.9    GTAAGCTGAAATAAACCCTTTCTTCCTCAGTCACTTTGGTCATGTTGTTTATCTTACACA
MC096-5LP_ref                   GTAAGCTGAAATAAACCCTTTCTTCCTCAGTCACTTTGGTCATGTTGTTTATCTTACACA

MC096-5LP-30243B-5_5639_63.1    GCAGTAGAAACGCTAACCCCTTCA----------------------------------GT
MC096-5LP-30243B-5_3302_36.9    GCAGTAGAAACGCTAACCCCTTCAATAACTTCGTATAATGTATGCTATACGAAGTTATGT
MC096-5LP_ref                   GCAGTAGAAACGCTAACCCCTTCAATAACTTCGTATAATGTATGCTATACGAAGTTATGT

MC096-5LP-30243B-5_5639_63.1    TACCGTCCTCAGGATATATTTTGGAAGCATGTTTGGGCTGCAAGCTGTTCATGGTTGTTC
MC096-5LP-30243B-5_3302_36.9    TACCGTCCTCAGGATATATTTTGGAAGCATGTTTGGGCTGCAAGCTGTTCATGGTTGTTC
MC096-5LP_ref                   TACCGTCCTCAGGATATATTTTGGAAGCATGTTTGGGCTGCAAGCTGTTCATGGTTGTTC

MC096-5LP-30243B-5_5639_63.1    TGGAGTTCTTTTCACGCCCCT
MC096-5LP-30243B-5_3302_36.9    TGGAGTTCTTTTCACGCCCCT
MC096-5LP_ref                   TGGAGTTCTTTTCACGCCCC-
```

MC096-3LP-1-30243B-5

```
MC096-3LP-30243B-5_843_34.2     GCTCCCACTTAGAAGCCTAAGGGCTGCTTAGTGTCCACGCCCACACACTTGATGACTGCC
MC096-3LP_ref                   GCTCCCACTTAGAAGCCTAAGGGCTGCTTAGTGTCCACGCCCACACACTTGATGACTGCC
MC096-3LP-30243B-5_1621_65.8    GCTCCCACTTAGAAGCCTAAGGGCTGCTTAGTGTCCACGCCCACACACTTGATGACTGCC

MC096-3LP-30243B-5_843_34.2     GCAGCAGGGTCTGTAAAACCTGGTATAACTTCGTATAATGTATGCTATACGAAGTTATGC
MC096-3LP_ref                   GCAGCAGGGTCTGTAAAACCTGGTATAACTTCGTATAATGTATGCTATACGAAGTTATGC
MC096-3LP-30243B-5_1621_65.8    GCAGCAGGGTCTGTAAAACCTGG----------------------------------TGC

MC096-3LP-30243B-5_843_34.2     GCATCCCACTATGTAGCTTGGTGCCCTTTTCTTTGTTACATTGTGATCATATAAAACCGG
MC096-3LP_ref                   GCATCCCACTATGTAGCTTGGTGCCCTTTTCTTTGTTACATTGTGATCATATAAAACCGG
MC096-3LP-30243B-5_1621_65.8    GCATCCCACTATGTAGCTTGGTGCCCTTTTCTTTGTTACATTGTGATCATATAAAACCGG

MC096-3LP-30243B-5_843_34.2     CTCACTGGCAGGCGATTTTG
MC096-3LP_ref                   CTCACTGGCAGGCGATTTTG
MC096-3LP-30243B-5_1621_65.8    CTCACTGGCAGGCGATTTTG
```

Applied StemCell, Inc.
521 Cottonwood Dr, Suite 111, Milpitas, CA 95035
Phone: (866) 497-4180 (US Toll Free); 408-773-8007 Fax: 408-773-8238
info@appliedstemcell.com www.appliedstemcell.com

MC096-5LP-1-30243B-8

```
MC096-5LP-30243B-8_5869_60.1     GTAAGCTGAAATAAACCCTTTCTTCCTCAGTCACTTTGGTCATGTTGTTTATCTTACACA
MC096-5LP-30243B-8_3889_39.9     GTAAGCTGAAATAAACCCTTTCTTCCTCAGTCACTTTGGTCATGTTGTTTATCTTACACA
MC096-5LP_ref                    GTAAGCTGAAATAAACCCTTTCTTCCTCAGTCACTTTGGTCATGTTGTTTATCTTACACA

MC096-5LP-30243B-8_5869_60.1     GCAGTAGAAACGCTAACCCCTTCA----------------------------------GT
MC096-5LP-30243B-8_3889_39.9     GCAGTAGAAACGCTAACCCCTTCAATAACTTCGTATAATGTATGCTATACGAAGTTATGT
MC096-5LP_ref                    GCAGTAGAAACGCTAACCCCTTCAATAACTTCGTATAATGTATGCTATACGAAGTTATGT

MC096-5LP-30243B-8_5869_60.1     TACCGTCCTCAGGATATATTTTGGAAGCATGTTTGGGCTGCAAGCTGTTCATGGTTGTTC
MC096-5LP-30243B-8_3889_39.9     TACCGTCCTCAGGATATATTTTGGAAGCATGTTTGGGCTGCAAGCTGTTCATGGTTGTTC
MC096-5LP_ref                    TACCGTCCTCAGGATATATTTTGGAAGCATGTTTGGGCTGCAAGCTGTTCATGGTTGTTC

MC096-5LP-30243B-8_5869_60.1     TGGAGTTCTTTTCACGCCCCT
MC096-5LP-30243B-8_3889_39.9     TGGAGTTCTTTTCACGCCCCT
MC096-5LP_ref                    TGGAGTTCTTTTCACGCCCC-
```

MC096-3LP-1-30243B-8

```
MC096-3LP-30243B-8_310_37.9      GCTCCCACTTAGAAGCCTAAGGGCTGCTTAGTGTCCACGCCCACACACTTGATGACTGCC
MC096-3LP_ref                    GCTCCCACTTAGAAGCCTAAGGGCTGCTTAGTGTCCACGCCCACACACTTGATGACTGCC
MC096-3LP-30243B-8_509_62.1      GCTCCCACTTAGAAGCCTAAGGGCTGCTTAGTGTCCACGCCCACACACTTGATGACTGCC

MC096-3LP-30243B-8_310_37.9      GCAGCAGGGTCTGTAAAACCTGGTATAACTTCGTATAATGTATGCTATACGAAGTTATGC
MC096-3LP_ref                    GCAGCAGGGTCTGTAAAACCTGGTATAACTTCGTATAATGTATGCTATACGAAGTTATGC
MC096-3LP-30243B-8_509_62.1      GCAGCAGGGTCTGTAAAACCTGG----------------------------------TGC

MC096-3LP-30243B-8_310_37.9      GCATCCCACTATGTAGCTTGGTGCCCTTTTCTTTGTTACATTGTGATCATATAAAACCGG
MC096-3LP_ref                    GCATCCCACTATGTAGCTTGGTGCCCTTTTCTTTGTTACATTGTGATCATATAAAACCGG
MC096-3LP-30243B-8_509_62.1      GCATCCCACTATGTAGCTTGGTGCCCTTTTCTTTGTTACATTGTGATCATATAAAACCGG

MC096-3LP-30243B-8_310_37.9      CTCACTGGCAGGCGATTTTG
MC096-3LP_ref                    CTCACTGGCAGGCGATTTTG
MC096-3LP-30243B-8_509_62.1      CTCACTGGCAGGCGATTTTG
```

Figure 1: The heterozygotes determined from PCR/NGS analysis of F0 founder 3024#3 B litter. The loxp sequence is highlighted in yellow. MC096, project #; LP, loxp; 30243B, the B litter from F0 founder 3024#3; -2, 5, 8, the heterozygous pup #.

Shipment

The mice when they are weaned at age of 3 weeks to be shipped to clients old are listed in the table 2. The female F0 founder 3024#3 will be bred to make one more litter as a backup.

Table 2 mice to be shipped

Mouse ID	DOB	gender	5 loxp	3 loxp
3024#3B#2	2/1/18	M	V	v
3024#3B#5	2/1/18	M	v	V
3024#3B#8	2/1/18	F	V	v

Prepared by:

James Luo, Ph.D.
Senior Scientist & Project Manager
Applied StemCell, Inc.
James.luo@appliedstemcell.com

Generation of gRNAs and DNA donors for mFermt2-cKO Models

Customer Information

**Project Title**	mFermt2-cKO
**Job Ticket**	MC096
**Client Name**	Ed Plow, Ph.D.
**Client Institute**	Cleveland Clinic
**Report Date**	11/22/2016

Summary

The goal of this project is to generate mouse Fermt2 conditional knockout mouse model (Fermt2-cKO). This can be achieved by inserting two LoxP sites in the introns flanking certain exons of Fermt2 gene (ENSEMBL Gene ID: ENSMUSG00000037712) to cover all 4 transcripts (splice variants). In such a model, those exons can be removed when the LoxP-inserted mice are crossed with Cre-expressing mice. To promote knockin of the LoxP sequences, double strand breaks (DSBs) will be made thought CRISPR/Cas9 system. Single guide RNA for each targeting site was prepared and validated for the efficiency in directing the cleavage in the mouse genome. Single stranded oligodeoxynucleotide (ssODN) donors were designed and generated for knockin of LoxP sequence according to the most active gRNA sequences. Qualified gRNAs, ssODNs and Cas9 protein were prepared for zygotic microinjection.

Selection, preparation, and validation of gRNAs for application of CRISPR/Cas9 system

1. **Strategy of guide RNA (gRNA) selection**
The criteria for gRNA selection are the high specificity and the close distance to the targeting site. The exons1 or 2 to exon 14 from Fermt2-001 gene transcripts (total 15 exons) are to be flanked by LoxP in order to remove all these flanked exons in the presence of Cre recombinase resulting in knockout of all 4 transcripts (splice variants). A LoxP sequence will be respectively inserted into 5'UTR or intron 1 and intron 14 where a DSB will be made through CRISPR/Cas9 approach to promote site specific knock-in. Multiple gRNA candidates targeting 5'UTR (Fermt2.5g63 and Fermt2.5g55) or intron 1 (Fermt2.5g8 and Fermt2.5g53) and intron 14 (Fermt2.3g68 and Fermt2.3g37) were selected. The sequences of the gRNA candidates and the Protospacer Adjacent Motif (PAM) are listed in **Table 1**.

Table 1. Guide RNA candidates for Fermt2 gene cKO

Name	Sequence	PAM
Fermt2.5g63	5'-CGCGCCCGCCTAGCGGACTT-3'	GGG
Fermt2.5g55	5'-CGGCTACGCGCGCACAACCA-3'	AGG

Fermt2.5g8	5′-CCCCTTCAGTTACCGTCCTC-3′	AGG
Fermt2.5g53	5′-ATCCTGAGGACGGTAACTGA-3′	AGG
Fermt2.3g68	5′-TACATAGTGGGATGCGCACC-3′	AGG
Fermt2.3g37	5′-CACTGGCAGGCGATTTTGCT-3′	TGG

2. Assessment of gRNA activity by next-generation sequencing (NGS)

After insertion of double-stranded oligos into a dual expression vector pBT-U6-Cas9-2A-GFP, each construct was transfected into mouse N2A cells. DNA was extracted from transfected cells and NGS was employed to analyze the percentage of non-homologous end joining (NHEJ) with the following primers (**Table 2**). The targeted regions and the picked primers for the tests were shown in the **Figure 1**. Fermt2.5g53 (targeting in intron1) and Fermt2.3g68 (targeting intron14) show great cutting efficiency which passes our QC standard of minimum efficiency (15%) (**Figure 2**). The candidate targeting sites 5g63 and 5g55 were not successfully tested due to the high percentage of G-C contents in the region which caused failure in sequencing, both sites were not employed for further experiments. The final strategy for making Fermt2 cKO mouse model is illustrated in **Figure 3**.

Table 2 Primers for deep sequencing

Primer ID	Sequence
Fermt2.5g.ds.F	5′-CACTCTTTCCCTACACGACGCTCTTCCGATCTGCATGCTTCCGTGCCATCTGCTTTA-3′
Fermt2.5g.ds.R	5′-GTGACTGGAGTTCAGACGTGTGCTCTTCCGATCTGCCTCTGGCTTTTGTGGATACCAGCA-3′
Fermt2.3g.ds.F	5′-CACTCTTTCCCTACACGACGCTCTTCCGATCTGCCTCTATGCCTCACCTCAGCCTTC-3′
Fermt2.3g.ds.R	5′–GTGACTGGAGTTCAGACGTGTGCTCTTCCGATCTACGGATGGTTGTGAGCCACCATGTG-3′

Illumina adapters in color. Red = Forward adapter. Blue = Reverse adapter. Black = base primer.

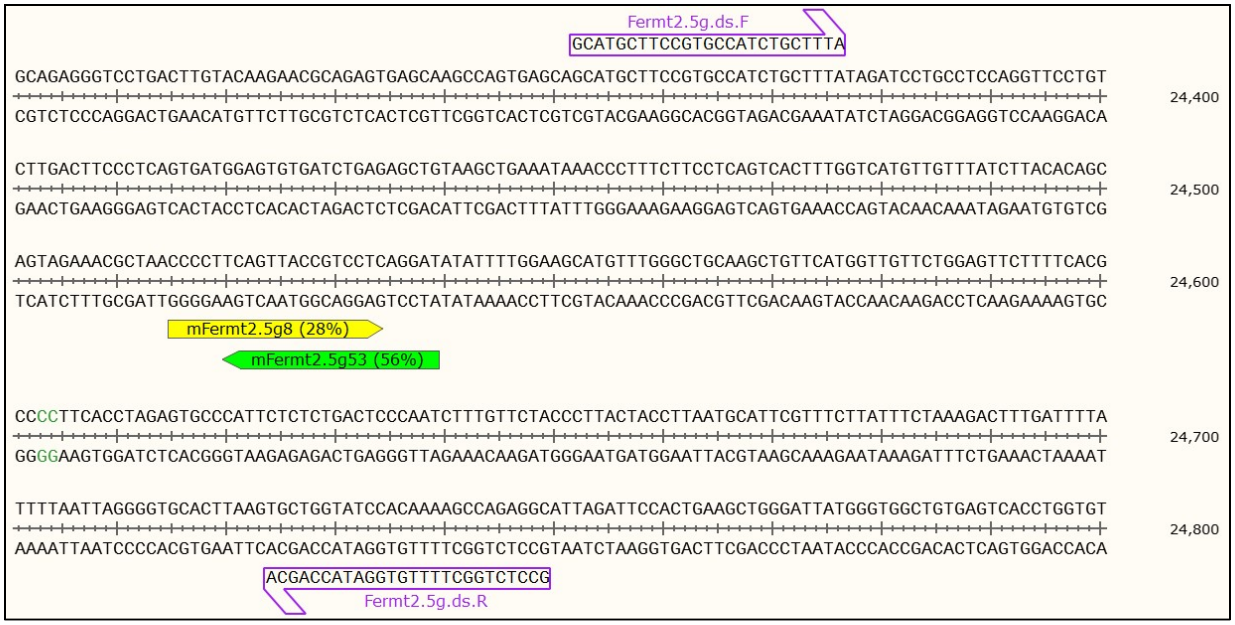

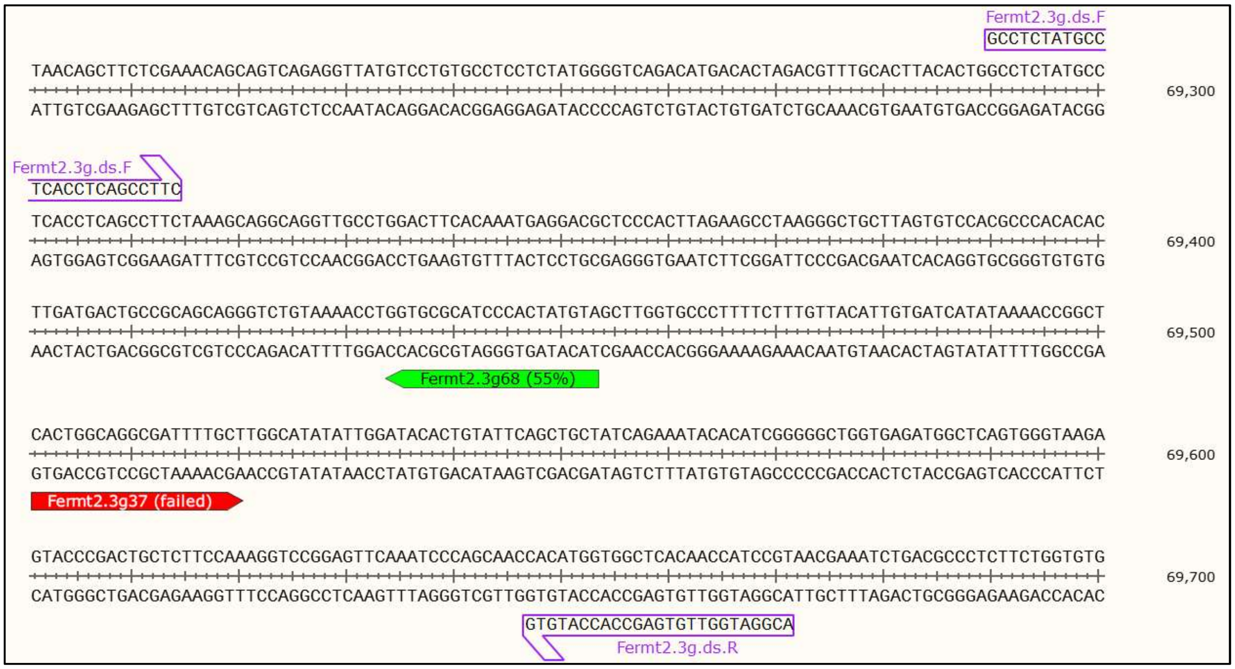

Figure 1. the targeted regions and the picked primers for the deep sequencing. Top panel, upstream in the intron 1; bottom panel, downstream in the intron 14.

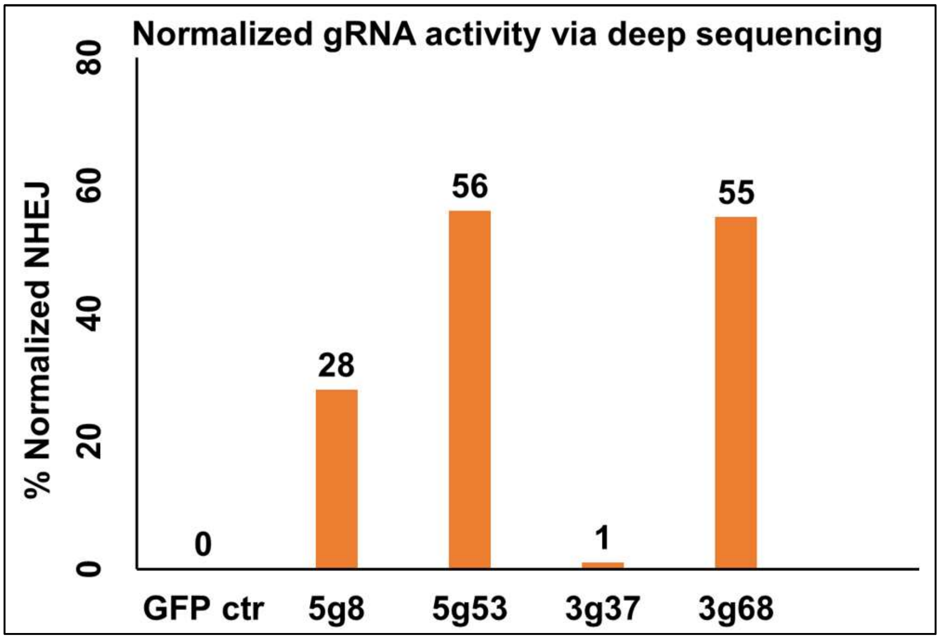

Figure 2. The activity of the selected gRNA measured by NHEJ% through deep sequencing

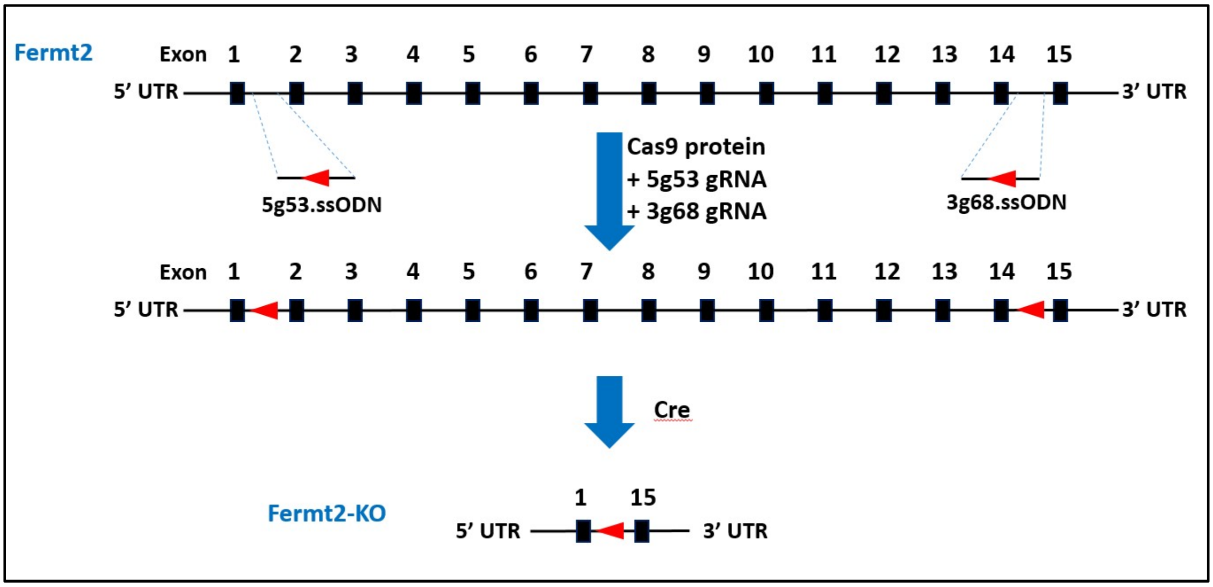

Figure 3. The final strategy to make Fermt2 gene cKO transgenic mouse model

3. Generation of single gRNA through *in vitro* transcription (IVT)

Based on the validated gRNAs Fermt2.5g53 and Fermt2.3g68 targeting sites, the forward primers Fermt2. 5g53.sgRNA_F or Fermt2. 3g68.sgRNA_F containing a leading T7 promoter sequence, a targeting site guide sequence, and a universal annealing oligo at 3' end as well as a reverse primer were synthesized (**Table 3**). With these primers, a plasmid pSpCas9 (BB)-2A-GFP (#48138, Addgene, or pX330) was applied as a template to make respective IVT template in a PCR reaction. The PCR products were gel-purified for IVT using MEGAshortscript T7 kit (Life Technologies, cat#: Am1354M). The IVT was performed with the mMESSAGE mMACHINE T7 ULTRA kit (Life Technologies, cat#: AM1345) according to the manual. IVT gRNAs were purified using MEGAclear kit (Life Technologies, cat#: AM1908M) and eluted in RNA elution buffer. The eluted RNA will be checked by agarose gel electrophoresis and quantitated by NanoDrop measurement (A260/280>=2.0).

Table 3. Primers used for T7- single gRNA RNA IVT

Name	Sequence
**Fermt2. 5g53.sgRNA_F**	5'-CGTTAATACGACTCACTATAGGATCCTGAGGACGGTAACTGAGTTTTAGAGCTAGAAATA-3'
**Fermt2. 3g68.sgRNA_F**	5'-CGTTAATACGACTCACTATAGGTACATAGTGGGATGCGCACCGTTTTAGAGCTAGAAATA-3'
**sgRNA.scaffold_R**	5'-AAAAAAGCACCGACTCGGTGCCAC-3'

Donor DNA design and preparation

1. Donor ssODN design and synthesis

In order to insert Lox P sites into the intron 1 and intron 14 of Fermt2 gene through endogenous homology-directed repair (HDR), two ssODNs containing 5' and 3' homologous arms flanking the Lox P sequence were designed for synthesis.

mFermt2.5g53.ssODN: LoxP; Target site; PAM

GTAAGCTGAAATAAACCCTTTCTTCCTCAGTCACTTTGGTCATGTTGTTTATCTTACACAGCAGTAGAAACGCTAACCCCTTCAATAACTTCGTATAATGTATGCTATACGAAGTTATGTTACCGTCCTCAGGATATATTTTGGAAGCATGTTTGGGCTGCAAGCTGTTCATGGTTGTTCTGGAGTTCTTTTCACGCCCC

mFermt2.3g68.ssODN: LoxP; Target site; PAM

GCTCCCACTTAGAAGCCTAAGGGCTGCTTAGTGTCCACGCCCACACACTTGATGACTGCCGCAGCAGGGTCTGTAAAACCTGGTATAACTTCGTATAATGTATGCTATACGAAGTTATGCGCATCCCACTATGTAGCTTGGTGCCCTTTTCTTTGTTACATTGTGATCATATAAAACCGGCTCACTGGCAGGCGATTTTG

Prepared by:
James Luo
Senior Scientist & Project Manager
Applied StemCell, Inc.
James.luo@appliedstemcell.com

Identification of F0 founders of mFermt2-cKO

Customer information

Project Title	mFermt2-CKO
Job Ticket	MC096
Client Name	Ed Plow, Ph.D.
Client Institute	Cleveland Clinic
Report Date	01/22/2017

Summary

CRISPR/Cas9 system was applied to generate mFermt2-cKO mouse models. According to the design (refer to the report1), a mixture of both upstream and downstream of gRNAs, both donor ssODN containing a loxP site, and recombinant Cas9 protein was microinjected into mouse zygotes that were transferred into pseudo-pregnant females to produce F0 pups. F0 pups were genotyped by PCR and sequencing to analyze the potentially modified targeting sites for LoxP insertion, INDEL, or wild type (wt) sequence. Twelve F0 pups was born on 1/4/17, their genotypes were determined (**Table 1**). The further steps (retargeting and 2nd round of injection) will be taken to produce the Fermt2 F0 founders.

Table 1: 1st round microinjection and pups born

gRNAs: 5g53 + 3g68 (ng/µl)	Donor DNA: 5'ssODN + 3' ssODN (ng/µl)	Cas9 protein (ng/µl)	Injection route	# embryos injected	# embryos/# recipient	# Pups born
50 + 50	125 + 125	100	cytoplasmic	175	137/5	12

Method - Identification of mFertm2-cKO mice

Tail tissues from F0 mice were collected and DNA extraction was performed individually. One set of PCR primers, “genotyping PR 5F/5R”, amplifying a 230 bp (wt) or 264 bp (LoxP) fragment in intron 1-2, and another set of PCR primers, “genotyping PR 3F/3R”, amplifying a 288 bp (wt) or 322 bp (LoxP) in intron 14-15 were used to identify mFermt2 mutation (**Table 2** and **Figure 1**). PCR products were submitted for sequencing to search for INDEL, LoxP, and/or wt sequence.

Table 2. Primers used in identification of Fermt2 mutants

Primer name	Primer sequence	Amplicon (bp)
**Genotyping PR 5F**	5′-CTTCCCTCAGTGATGGAGTGTGATCTGAG-3′	230/264
**Genotyping PR 3R**	5′-GGAGTCAGAGAGAATGGGCACTCTAGGTG-3′	
**Genotyping PR 3F**	5′-CTAAAGCAGGCAGGTTGCCTGGAC-3′	288/322
**Genotyping PR 3R**	5′-CTCTTACCCACTGAGCCATCTCACC-3′	

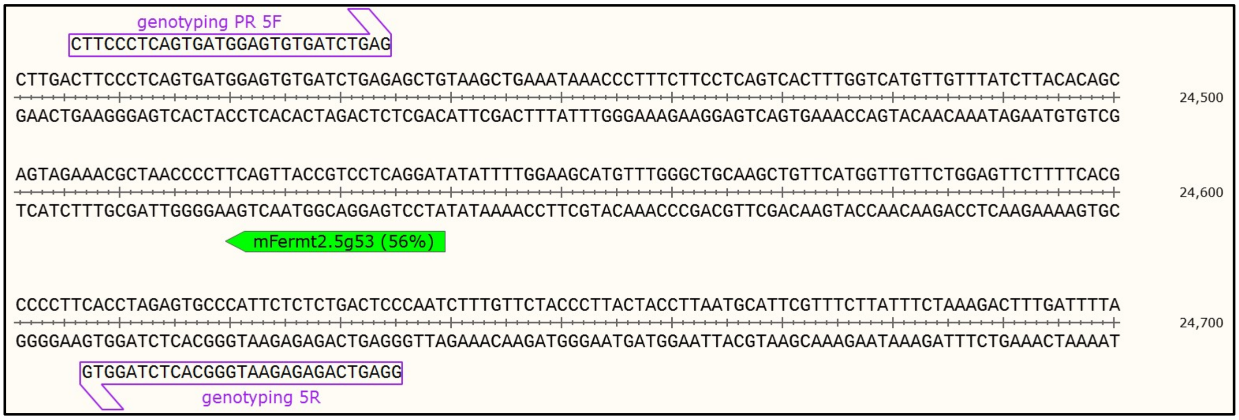

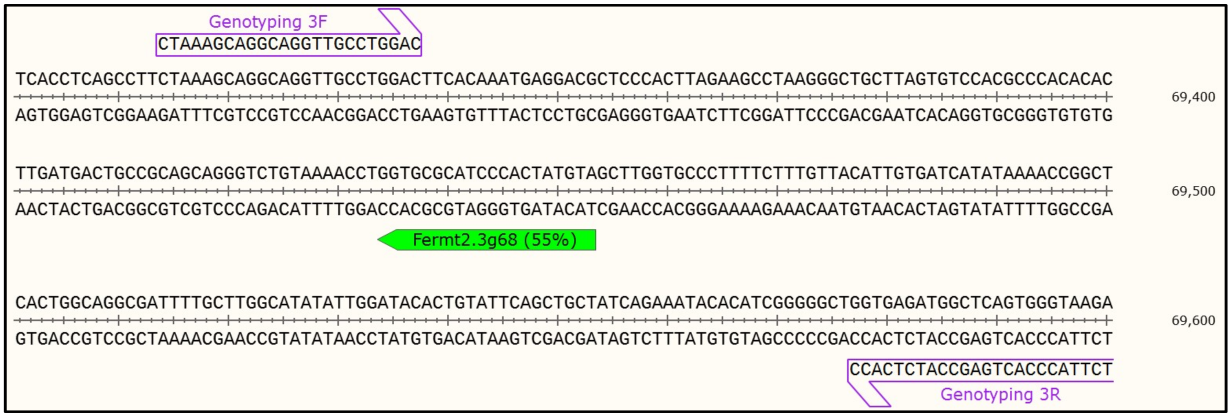

Figure 1. PCR-sequencing scheme to identify mutants in mouse Fermt2 gene. Top panel, intron 1-2 site; Bottom panel, intron 14-15 site; targeting sites were featured with green arrows containing gRNA information.

Results – genotypes of F0 mice

The 12 pups (2252#1 to 2252#6 and 2254#1 to 2254#6) were genotyped with PCR amplification of the intron 1-2 and intron 14-15 with the primers listed in the **table 2**. As shown in the figure 2, at intron 1-2, the bands amplified from pups 2252#1, 2252#6, 2254#5 are above the band from wt control which suggests that these pups might have loxp insertion; in the same scenario, at intron 14-15, the pups 2252#3, 2252#4, 2252#5, and 2254#4 might contain loxp insertion. To confirm the gel prediction, the PCR products were further subjected to Sanger sequencing (**Table 3**).

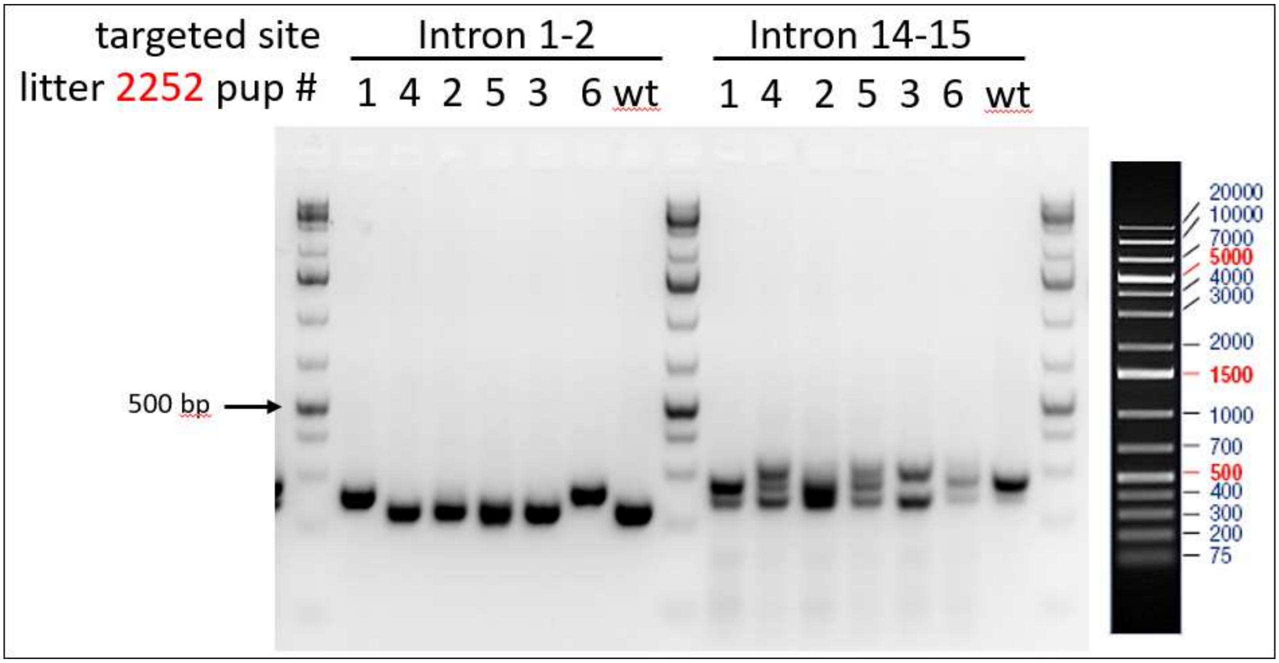

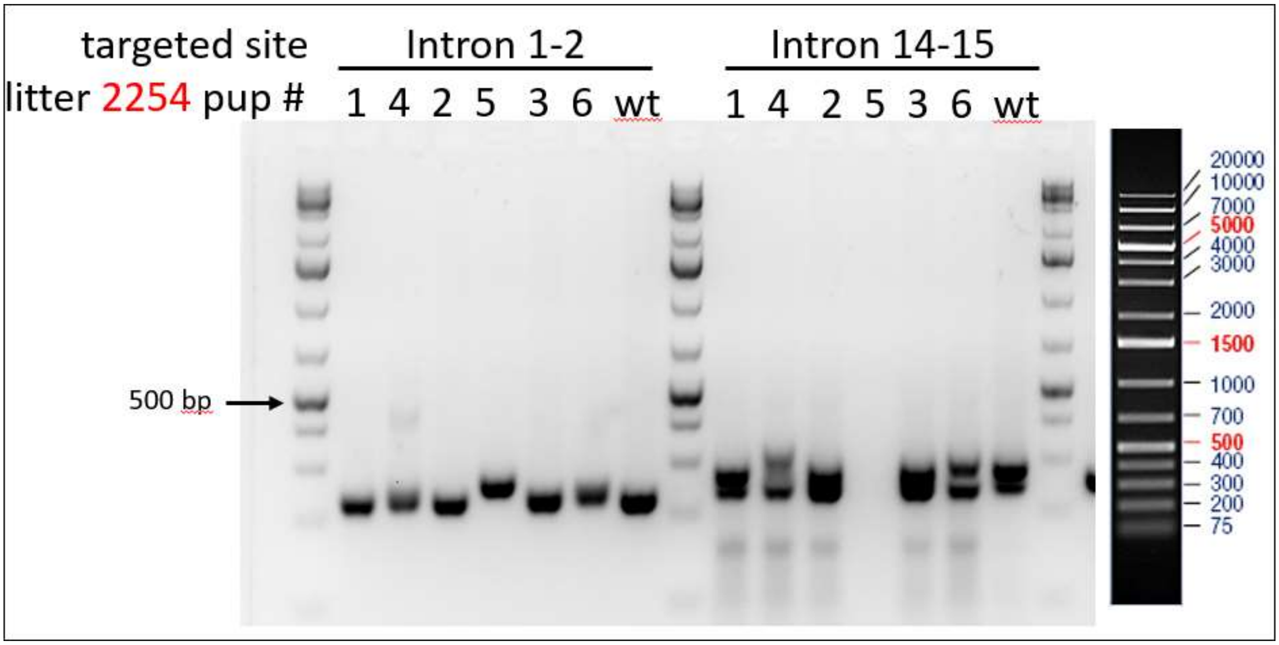

Figure 2. Electrophoresis of the PCR products amplified from the genomic DNA of F0 pups at mFermt2 gene intron 1-2 and intron 14-15 regions prior to sequencing analysis

Table 3. summary of the results from sanger sequencing

2252#	Intron1-2	Intron 14-15
1	loxp	Indel
2	Indel	Indel
3	Indel	LoxP
4	Indel	Indel
5	Indel	Indel
6	loxP/Indel	wt
2254#	Intron1-2	Intron 14-15
1	Indel	indel
2	wt	indel
3	Indel	indel
4	Indel	Indel
5	loxp	Indel
6	Indel	partial loxp

Applied StemCell, Inc.
521 Cottonwood Dr, Suite 111, Milpitas, CA 95035
Phone: (866) 497-4180 (US Toll Free); 408-773-8007 Fax: 408-773-8238
info@appliedstemcell.com www.appliedstemcell.com

Keep the F0 mice with one loxp insertion for re-targeting

Table 4. the F0 with one loxp inserted

Mouse ID	DOB	gender	5 loxp	3 loxp
2252#1	1/4/17	M	V	
2252#3	1/4/17	F		V
2252#6	1/4/17	F	V	
2254#5	1/4/17	F	V	

The F0 females with one loxp insertion (2252#3, 2252#6, and 2254#5) will be superovulated at age of 4 weeks (2/1/17) for re-targeting by microinjection of the eggs produced from these F0 positive mice mating with wt males.

Prepared by:

James Luo, Ph.D.

Senior Scientist & Project Manager

Applied StemCell, Inc.

James.luo@appliedstemcell.com

Production and identification of 5'+3' loxp F0 founders

Customer information

Project Title	mFermt2-cKO
Job Ticket	MC096
Client Name	Ed Plow, Ph.D.
Client Institute	Cleveland Clinic
Report Date	06/11/2017

Summary

In the 1st round of experiment, delivery of both upstream (5') and downstream (3') modification reagents was done in order to simultaneous modification of both sites, however, this strategy did not work. In most cases (10/12), a F0 mouse contains two indels or one indel/one Loxp at two targeting sites, and these mice are useless intermediate products. Therefore, sequential modifications of upstream (5') and downstream (3') through separate injection of 5' reagents and then 3' reagents were carried out in this report to increase each site knock-in efficiency and successfully generate both 5' and 3' loxp-inserted F0 founders.

First step: targeting at 5' locus with wild type embryos

Microinjection of 5g53 gRNA/Cas9 protein and 5' ssODN was done to insert a loxp at upstream (5') locus (Table 1). Genomic DNA was extracted from individual F0 pups and used as templates for PCR analysis (Table 2 and Figure1). PCR products were purified for sequence analysis, and the data was summarized in Table 3. The mice with 5' loxp inserted was saved for retargeting.

Table 1: 1st step 5' microinjection and pups born

gRNAs: 5g53 (ng/μl)	Donor DNA: 5'ssODN (ng/μl)	Cas9 protein (ng/μl)	Injection route	# embryos injected	# embryos/# recipient	# Pups born
100	250	100	cytoplasmic	95	74/3	13

Applied StemCell, Inc.
521 Cottonwood Dr, Suite 111, Milpitas, CA 95035
Phone: (866) 497-4180 (US Toll Free); 408-773-8007 Fax: 408-773-8238
info@appliedstemcell.com www.appliedstemcell.com

Table 2. PCR primers used in 5' F0 genotyping

Primer name	Primer sequence	Amplicon (bp)
**Genotyping PR 5F**	5'-CTTCCCTCAGTGATGGAGTGTGATCTGAG-3'	230/264
**Genotyping PR 3R**	5'-GGAGTCAGAGAGAATGGGCACTCTAGGTG-3'	

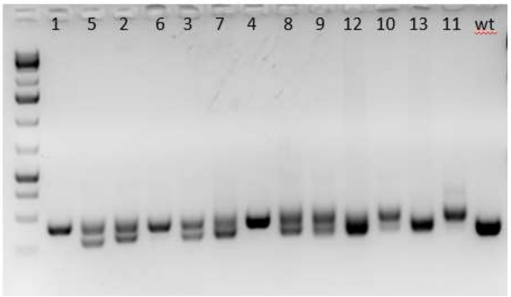

Figure 2 electrophoresis of PCR products from 5' F0 pups

Table 3 sequence analysis of genotypes of the 5' F0 pups

2437#	Gender	Genotypes	Retargeting
**1**	M	correct loxp	Yes
**2**	M	correct loxp	Yes
**3**	M	correct loxp	Yes
**4**	M	correct loxp	Yes
**5**	M	correct loxp	yes
**6**	F	correct loxp	Yes
**7**	F	correct loxp	Yes
**8**	F	correct loxp	lost
**9**	F	correct loxp	Yes
**10**	F	Partial loxp	No
**11**	F	correct loxp	Yes
**12**	F	indel	No
**13**	F	indel	No

Second step: targeting at 3' locus with 5' loxp-inserted embryos

F0 mice containing 5' loxp (5' F0 founders) either males or females are mated with wild type mice to produce embryos for further injection of 3' reagents in order to insert 3' loxp in the genome. Female 5' F0 founders (2437# 6, 7, 9, 11) at 3-4wk old were superovulated and mated with wild type males to produce embryos containing 5' loxp for the earliest injection of 3' reagents (3g68 gRNA/Cas9 protein, 3'ssODN) (Table 4). The pups produced from the 2nd step injection were

Applied StemCell, Inc.
521 Cottonwood Dr, Suite 111, Milpitas, CA 95035
Phone: (866) 497-4180 (US Toll Free); 408-773-8007 Fax: 408-773-8238
info@appliedstemcell.com www.appliedstemcell.com

genotyped to search for both 5' loxp and 3' loxp in the genome by PCR and sequencing analysis (Tables 5 and 6). The potential 5'+3' F0 founders were listed in table 7.

Table 4: 2nd step 3' microinjection and pups born

gRNAs: 3g68 (ng/µl)	Donor DNA: 3'ssODN (ng/µl)	Cas9 protein (ng/µl)	Injection route	# embryos injected	# embryos/# recipient	# Pups born
100	250	100	cytoplasmic	76	59/2	10

Table 5. Primers used in identification of Fermt2 mutants

Primer name	Primer sequence	Amplicon (bp)
Genotyping PR 5F	5'-CTTCCCTCAGTGATGGAGTGTGATCTGAG-3'	230/264
Genotyping PR 3R	5'-GGAGTCAGAGAGAATGGGCACTCTAGGTG-3'	
Genotyping PR 3F	5'-CTAAAGCAGGCAGGTTGCCTGGAC-3'	288/322
Genotyping PR 3R	5'-CTCTTACCCACTGAGCCATCTCACC-3'	

Table 6 Next generation sequencing (NGS) analysis of 2nd step injection-derived pups

5'+3' F0 founders	Gender	Pup 2580#	5' Loxp	Count %	3' Loxp	Count %
**v**	**Male**	**1**	+	**18.9**	+	**25**
x	male	2	-		+	21.7
x	Male	3	+	22.4	+/insertion	8.6
x	Male	5	+	19.7	-	
x	Male	5	+	21.4	-	
x	Female	6	-		+	29.9
x	Female	7	-		-	
**v**	**Female**	**8**	+	**23.3**	+	**18.7**
**v**	**Female**	**9**	+	**21.6**	+	**4.5**
x	Female	10	+	16.4	-	

Next step: selection of the *cis* loxp insertion in the genome (F1)

Table 7. 5'+ 3' loxp F0 founders

Mouse ID	DOB	gender	5 loxp	3 loxp
2580#1	5/17/17	M	V	v
2580#8	5/17/17	F	v	V
2580#9	5/17/17	F	V	v

To select *cis* loxp in the genome of 5'+3' loxp F0 founders, they will be mated with wild type mice to produce pups (F1) for further analysis. The F1 pups containing both 5' and 3' loxp will be the heterozygous cKO founders.

Prepared by:

James Luo, Ph.D.

Senior Scientist & Project Manager

Applied StemCell, Inc.

James.luo@appliedstemcell.com
