# Supplementary material for: Targeted Deletion of Kindlin-2 in Mouse Mammary Glands Inhibits Tumor Growth, Invasion, and Metastasis Downstream of a TGF-β/EGF Oncogenic Signaling Pathway
Source: Cancers (Basel). 2022 Jan 27;14(3):639. doi: 10.3390/cancers14030639 (PMC8833458; doi:10.3390/cancers14030639)
Supplement: Supplementary file 1 [file cancers-14-00639-s001.zip › cancers-1567837-supp-final/cancers-1567837-supp-final.pdf]

Supplementary Materials

Targeted Deletion of Kindlin-2 in Mouse Mammary Glands Inhibits Tumor Growth, Invasion and Metastasis Downstream of a TGF-β/EGF Oncogenic Signaling Pathway

Wei Wang, Priyanka S. Rana, Akram Alkrekshi, Katarzyna Bialkowska, Vesna Markovic, William P. Schiemann, Edward F. Plow, Elzbieta Pluskota, Khalid Sossey-Alaoui

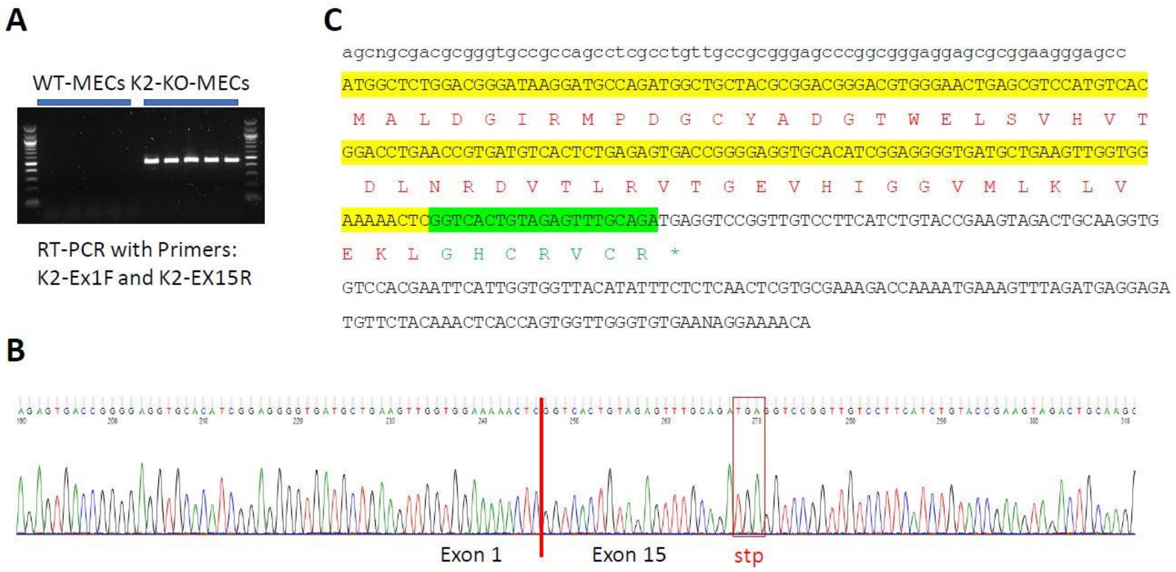

Figure S1. Cloning and sequencing of the Exon1-Exon15 RT-PCR product. Total RNA was isolated from WT or K2-/- MECs, transcribed to cDNA using BioRAD superscript cDNA synthesis kit. cDNA was then PCR amplified using RT-PCR primers K2-EX1F and K2-EX15R. The PCR reaction was resolved by agarose gel electrophoresis. I pictogram is shown in (**A**). The ~420 bp PCR product was gel purified and sequenced. A trace of the Sanger sequence is shown in (**B**), where a sequence junction between Exon 1 and Exon 15 is identified by a vertical red line. A downstream stop codon (stp) is also shown (red box). **(C)** *In silco* translation of the RT-PCR sequence shows the translated exon 1 of Kindlin-2 (Yellow highlighted nucleotide sequence) and the corresponding protein sequence (red amino acid sequence) that starts with a Met and ends with a Lys. As a result of the deletion, Exon1 became fused to exon15 where the open reading frame continues in Exon 15 (Green amino acid sequence) until it hits a premature stop codon (*). The result a very short amino acid sequence (59 amino acid peptide) that function of which, if any is not known. In most cases, these short premature mRNA sequences are subjected non-sense mediated decay degradation and may never get the chance to be translated into proteins.

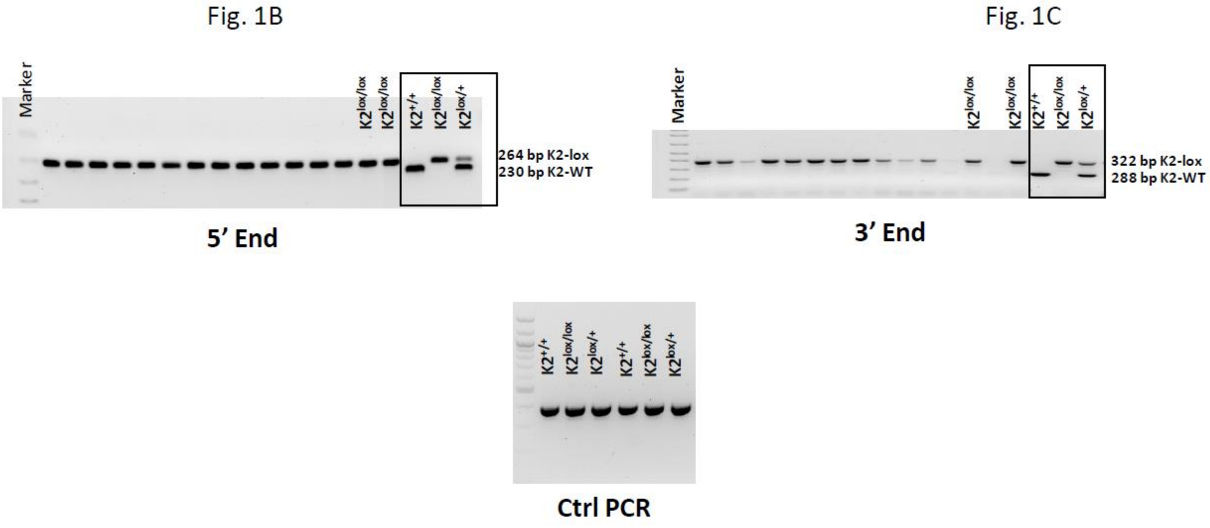

Figure S2. Uncropped full blot for Figure 1.

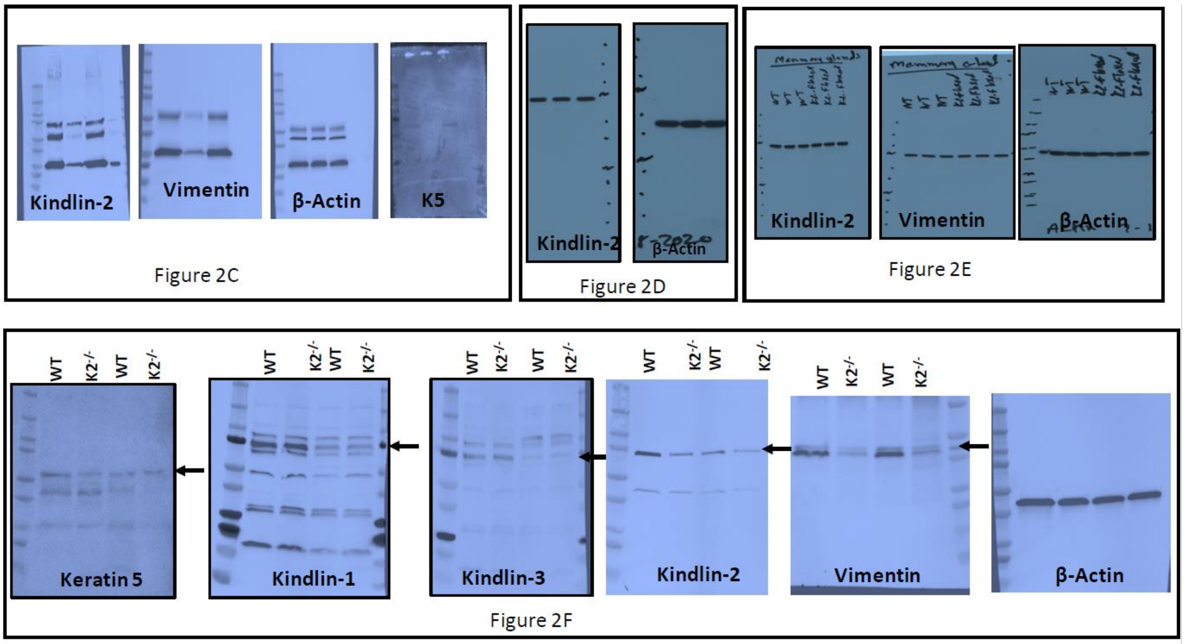

Figure S3. Uncropped full Western blot for Figure 2.

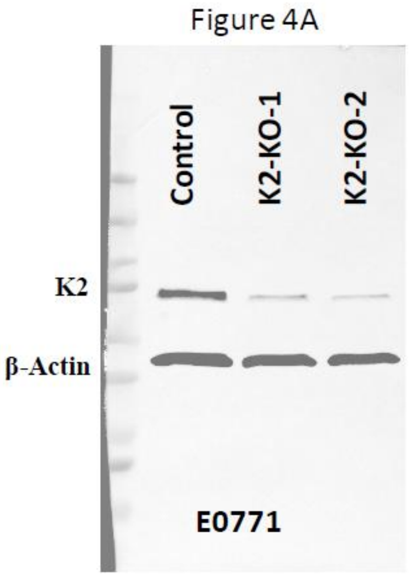

Figure S4. Uncropped full Western blot for Figure 4A.

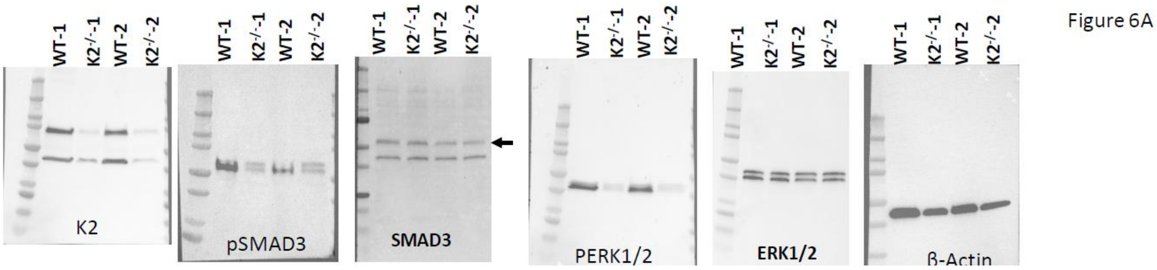

Figure S5. Uncropped full Western blot for Figure 6A.

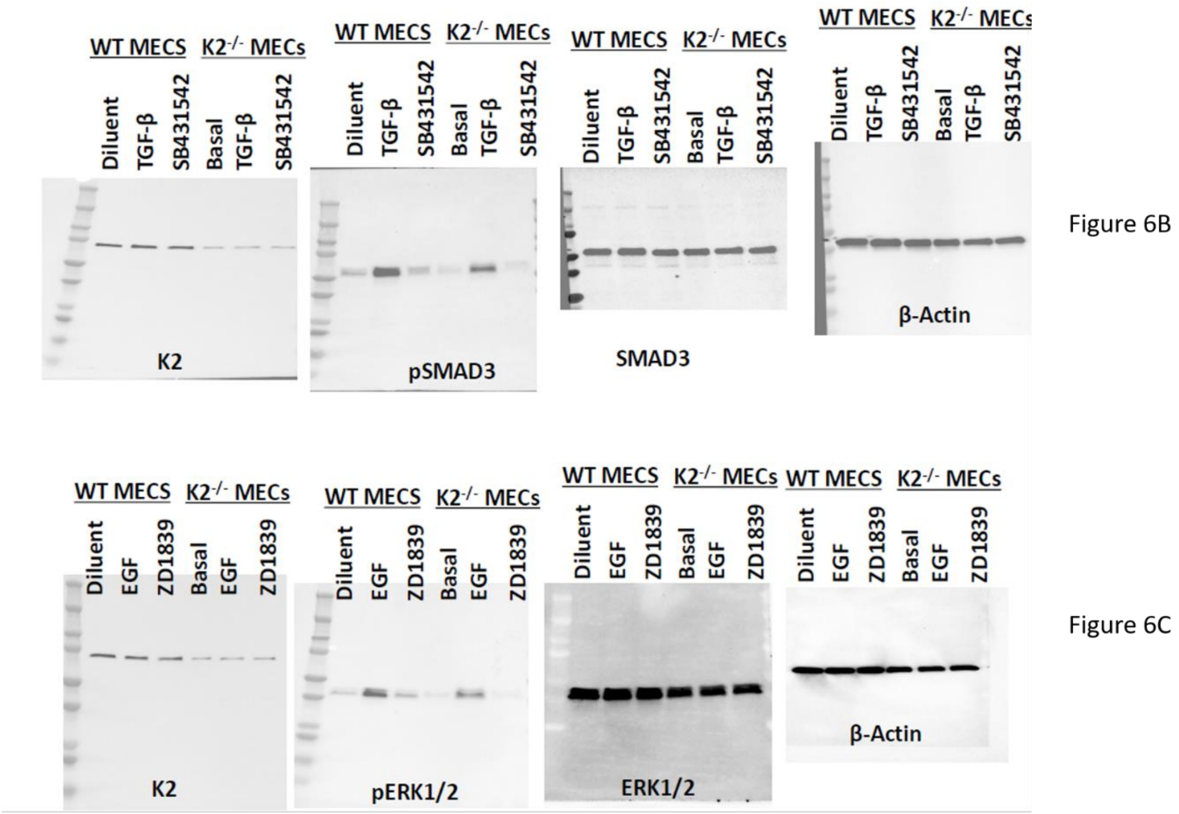

Figure S6. Uncropped full Western blot for Figure 6B,C.

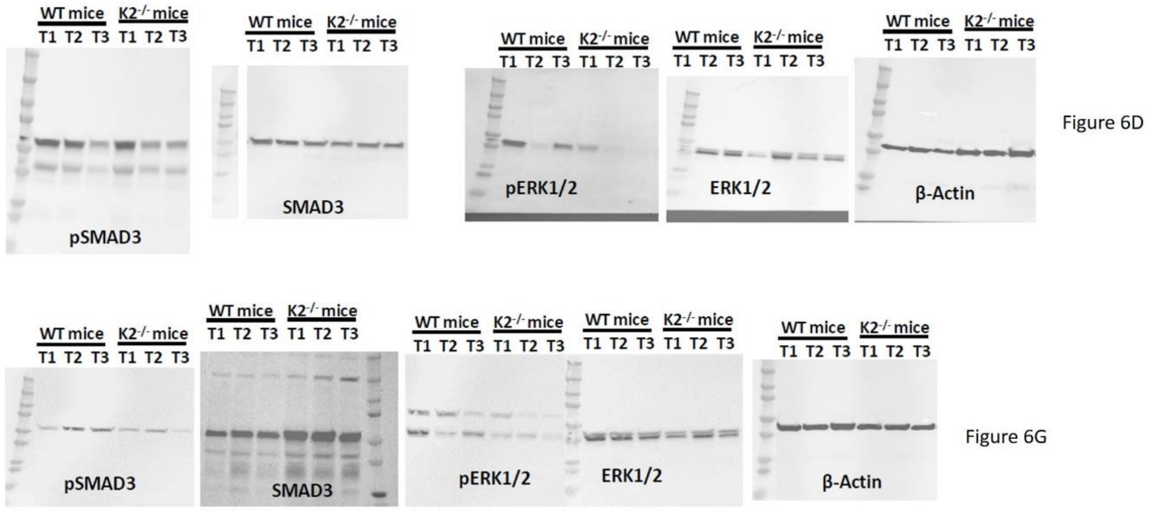

Figure S7. Uncropped full Western blot for Figure 6D,G.

© 2021 by the authors. Licensee MDPI, Basel, Switzerland. This article is an open access article distributed under the terms and conditions of the Creative Commons Attribution (CC BY) license (http://creativecommons.org/licenses/by/4.0/).
